# The effect of dietary weight‐loss interventions on the inflammatory markers interleukin‐6 and TNF‐alpha in adults with obesity: A systematic review and meta‐analysis of randomized controlled clinical trials

**DOI:** 10.1111/obr.13910

**Published:** 2025-03-16

**Authors:** Cate Bulmer, Alison Avenell

**Affiliations:** ^1^ Health Services Research Unit University of Aberdeen UK

**Keywords:** diet, inflammation, inflammatory markers, multimorbidity, obesity, weight loss

## Abstract

**Background:**

A chronic inflammatory state characterizes a wide range of diseases for which obesity is a risk factor. Weight loss could reduce levels of circulating inflammatory markers potentially reducing the incidence of associated diseases and improving response to treatment. However, dietary weight loss studies have reported inconsistent effects on serum inflammatory makers and the long‐term effects are unknown.

**Objective:**

To systematically review randomized controlled trials and analyze any differences in serum interleukin‐6 and tumor necrosis factor‐alpha between adults with obesity achieving weight loss through dietary intervention compared to those receiving none or standard care.

**Methods:**

Studies were identified by searching databases from 1966 to November 2024. Randomized controlled trials with at least 12 months' follow‐up were included in this systematic review and meta‐analysis with an assessment of Cochrane risk of bias version 1.

**Results:**

Twelve eligible studies were included. No trials reported a significant effect of weight loss on circulating tumor necrosis factor‐alpha, whilst studies achieving greater than 5% weight loss significantly reduced circulating interleukin‐6 in adults with obesity.

**Conclusion:**

Weight loss interventions achieving and maintaining greater than 5% weight loss appear to be required to reduce circulating interleukin‐6 levels in adults with obesity.

**Abbreviations**
BMI: Body Mass Index
WAT: White Adipose Tissue
TNF‐α: Tumor Necrosis Factor‐Alpha
IL‐6: Interleukin‐6
RCT: Randomized Controlled Trial
CVD: Cardiovascular Disease
SD: Standard deviation
MD: Mean difference
BP: Blood pressure
sTNFR: TNF‐α soluble receptors

## 1 INTRODUCTION

There is increasing evidence that a plethora of diseases are associated with obesity.^1^ Cross‐sectional analysis reveals that with every increase in the BMI (body mass index) category the prevalence of multimorbidity increases.^2^ Multimorbidity, the “coexistence of two or more long‐term medical conditions or diseases” is strongly associated with health service utilization, accounting for more than half of UK general practice consultations and hospital admissions.^3^, ^4^ Recently published findings from the UK Biobank showed that 55 year‐olds living with obesity have the same proportion of complex multimorbidity as healthy‐weight 75 year‐olds.^5^ In absolute terms, the working‐age UK population currently lives with more multi‐morbidity than the elderly, creating demands for healthcare far outstripping the National Health Service's resources.^6^

Chronic “low grade” inflammation is considered to play a role in the pathophysiology of a wide range of diseases, many associated with obesity.^7^ Once thought to function principally for the storage of energy as triacylglycerols, today white adipose tissue (WAT) is understood to be a dynamic and complex multifunctional organ, integrated with and regulating whole‐body physiology.^8^ Whereas lean WAT predominantly exists in an anti‐inflammatory state, it is proposed that pathological WAT expansion results in loss of homeostatic balance, accompanied by increasing production of pro‐inflammatory adipokines, such as tumor necrosis factor‐alpha (TNF‐α) and interleukin‐6 (IL‐6), characterizing obesity as a condition of chronic low‐grade inflammation.^9^

As well as a risk factor for developing disease, obesity influences disease severity, response to treatment, and prognosis for conditions from depression to cancer.^10^ For example, a trial of a dietary weight loss intervention for patients with inflammatory arthritis initiating TNF‐α blocker therapy found those who achieved 5% to 10% weight loss were three times more likely to experience minimal disease activity compared to those with <5% weight loss.^11^ Obesity is associated with increasing severity, hospitalization, and mortality from acute COVID‐19 infection; an association that continues after the initial infection as patients with obesity are less likely to have recovered at 12 months.^12^, ^13^ Circulating IL‐6 is closely associated with severe COVID‐19 infection and IL‐6 antagonists e.g. tocilizumab, have been shown to reduce mortality.^14^, ^15^ Inflammatory profiling of patients with long‐COVID has shown increased IL‐6 to be associated with greater severity of physical and mental impairment at five months post infection.^12^ The potential for weight loss interventions to reduce long Covid‐19 symptoms is being studied in the current ReDIRECT trial.^16^

Bariatric surgery is associated with marked decreases in inflammatory cytokines.^17^ This degree of weight loss is not indicative of the modest long‐term weight loss achievable by most, who may not have access to or choose a surgical treatment. Despite obesity affecting almost a third of the UK population, only 4035 people in England underwent a surgical intervention in 2021/22.^18^

Randomized controlled trials (RCTs) investigating the effect of dietary weight loss interventions on serum IL‐6 and TNF‐α concentrations in people with obesity lasting at least three months have shown conflicting results, reporting no effect, as well as decreases and increases compared to control.^19^, ^20^, ^21^, ^22^ These contradictory results may be due to heterogeneity in the age and gender of participants, the range of underlying pathology, and the degree of weight loss attained. Both aging and menopause are associated with higher circulating inflammatory molecules, as are many chronic diseases, whilst it remains unclear what degree of weight loss may significantly reduce IL‐6 and TNF‐α.^20^, ^23^

In general, weight loss studies lasting a year or more predominantly show initial weight loss followed by a plateau and progressive regain for the majority of participants.^24^ Trials reporting the effect of short‐term weight loss interventions on circulating inflammatory markers make it difficult to distinguish if changes seen are due to weight loss per se or energy restriction. Nor do they show the effect of weight maintenance or regain which are clinically relevant to disease prevention, treatment, and outcome.

This systematic review and meta‐analysis were performed to analyze the effect of achieving weight loss through dietary interventions on serum IL‐6 and TNF‐α in adults with obesity with at least 12 months' follow‐up.

## 2 METHODS

### 2.1 Protocol and registration

The review followed the Preferred Reporting Items for Systematic Reviews and Meta‐Analyses (PRISMA) guidelines,^25^ with a protocol registered a priori (PROSPERO CRD42022320357).

### 2.2 Study selection

Eligible studies were RCTs comparing a dietary weight‐loss intervention, with or without exercise, to standard care or exercise only with at least 12 months of follow‐up. Eligible participants were adults (mean baseline age ≥ 18 years) with a mean BMI at baseline ≥ 30 kg/m^2^. Participant exclusion criteria were age <18 years; weight loss medication trials; anti‐TNF‐α and anti‐IL‐6 biologic therapy; weight loss surgery; participants all with pregnancy, breastfeeding, HIV infection, or cancer. The primary outcomes were differences in serum IL‐6 (pg/ml) and/or TNF‐α (pg/ml), and body weight (kg).

### 2.3 Literature searching

A systematic literature search of the Medline (Ovid) and Embase (Ovid) databases was conducted initially from inception to June 2023 (Figure S1). A full‐text search of a clinical trial register of long‐term weight management trials held at the Health Services Research Unit, University of Aberdeen from 1966 to 2017 was hand‐searched by AA with the help of SA for trials with relevant outcomes (since IL‐6 and TNF‐α may not appear in abstracts in databases). All database citations were screened for duplicates and the first three hundred titles and abstracts were assessed against inclusion/exclusion criteria independently by CB and AA. As no discrepancies were found CB screened the remaining titles and abstracts. Abstracts matching inclusion criteria that reported data outcomes at six months were included for full‐text review and read in detail to determine if any data were from one year or more by CB and checked by AA. Any disagreements were resolved by consensus. The search was updated in November 2024 with both authors independently screening the additional titles and abstracts. In addition, a manual search of the reference lists of eligible studies was conducted for relevant articles.

### 2.4 Data extraction

Data extraction was undertaken by CB and checked by AA, with differences resolved by discussion. Extracted data included: author, country, inclusion and exclusion criteria, number of participants, sex and mean age, intervention description and duration, and loss at follow‐up. Data pertaining to mean weight (kg), BMI, serum IL‐6, and TNF‐α at baseline and mean change at follow‐up were extracted as well as percentage change. If mean change was not stated it was calculated from the difference between mean outcome and baseline, as was percentage change if not presented. For weight, if not available, standard deviations (SD) were calculated from 95% Confidence Intervals or standard errors, or when these were not presented a prior regression equation.^26^

### 2.5 Quality assessment

Quality of evidence was assessed independently by both reviewers using the Cochrane Risk of Bias Tool Version 1.^27^ Studies were categorized as having either low, high, or unclear levels of bias for each of the domains; differences were resolved through discussion.

### 2.6 Data synthesis

When trials had more than two arms, trial arms with both a diet and exercise component were compared to exercise‐only arms, enabling isolation of the effects of dietary changes. Diet‐only arms were compared to control groups. Study data were summarized descriptively and presented in outcome tables. RevMan Web Version 5.5.0 was used for meta‐analysis where data were suitable for pooled analysis for weight. Pooled results for continuous outcomes were presented as mean difference (MD), using a continuous inverse variance method, and 95% CI. Heterogeneity was examined using the I^2^ statistic; I^2^ > 50% was classified as high heterogeneity. A random effects model was used due to expected high heterogeneity (I^2^ > 50%). Publication bias was assessed by visual inspection of the funnel plot for outcomes. Sensitivity analyses were performed for blinding of outcome assessment (low/unknown vs high risk of bias).

## 3 RESULTS

### 3.1 Search results

Searches yielded 4531 citations after duplicates were removed, resulting in 58 full‐text articles for detailed assessment (Figure S2). Bibliographies of these were searched identifying a further four studies. In total 12 studies were included. One author was contacted for further data which were kindly provided.^28^

### 3.2 Participant characteristics

Overall, 2805 individuals participated in 12 RCTs, with sample sizes ranging from 30 to 593 published between 2003 and 2022. Six trials were carried out in the USA,^29^, ^30^, ^31^, ^32^, ^33^, ^34^ two in the UK,^35^, ^36^ and Italy,^37^, ^38^ and one each in Finland,^39^ and Spain.^28^ Inclusion criteria covered ages from 20 years upwards. Five of the studies focused specifically on older adults.^28^, ^29^, ^30^, ^32^, ^33^ Two studies included only healthy women based on pre or post‐menopausal status,^34^, ^38^ and one study men with erectile dysfunction.^37^ The remaining studies included people with osteoarthritis,^30^, ^32^ impaired glucose tolerance,^31^, ^36^, ^39^ type 2 diabetes mellitus,^35^ or cardiometabolic disease.^28^, ^29^, ^33^ Women accounted for around two‐thirds of participants in the majority of studies. Mean baseline BMI ranged from 30.5 kg/m^2^ to 36.9 kg/m^2^. There were no reported significant differences in baseline characteristics between intervention and control groups in any of the studies (Table S1).

### 3.3 Trial design

Along with inflammatory biomarkers, outcomes varied widely including measures of insulin resistance and cardiovascular risk,^38^ endothelial and erectile function,^37^ knee joint compressive force,^32^ visceral adipose tissue,^33^ and frailty.^28^ Six papers were ancillary studies with the original study primary outcomes including; physical function and disability,^30^ serum estrone,^34^ diabetes incidence,^39^ mobility,^29^ fasting blood glucose and insulin resistance,^31^ HbA1c and BP^35^ and weight.^36^ All studies measured IL‐6, whilst three also reported TNF‐α.

Six of the twelve studies consisted of two arms comparing a dietary weight loss intervention to a control group.^28^, ^31^, ^36^, ^37^, ^38^, ^39^ Four trials randomized to one of three arms, and two had four arms ‐ these included weight loss with or without exercise, exercise only and control groups.^29^, ^30^, ^32^, ^33^, ^34^, ^35^ From these, fourteen sets of comparison groups were extracted for analysis; five comparing diet and exercise to exercise only, six compared diet and exercise to control (since the effect of exercise could not be removed), and the remaining three diet to control.

Four trials had a six‐month weight loss intervention followed by a maintenance phase lasting a further 6, 12, or 18 months, three of which reported outcomes at the end of each phase,^28^, ^29^, ^30^ the fourth upon completing the maintenance phase only.^34^ Eight of the studies consisted of an intervention phase only, lasting 12,^(^ ^31^, ^33^, ^35^, ^39^ ^)^ 18,^32^ 24,^37^, ^38^ or 36^36^ months. Of these five reported outcomes at 6 months as well as upon intervention completion.^32^, ^33^, ^35^

### 3.4 Types of dietary intervention and control

The aim of the dietary interventions was weight loss through calorie restriction, either based on gender and weight or personalized to individual energy expenditure. None of the interventions restricted intake to less than 1100 kcal/day. The dietary macronutrient content did not particularly vary between the studies with half specifying a diet based on approximately 50% to 60% carbohydrates, 15% to 20% proteins, and less than 30% total fat, with less than 10% saturated fat.^28^, ^32^, ^33^, ^37^, ^38^, ^39^ Two specified fat content (<30%) only,^30^, ^34^ another was based on the US Department of Agriculture MyPyramid Food Guidance System^29^ and the remaining two were not prescriptive beyond ‘healthy eating’ but aimed to produce weight loss.^35^, ^36^ Three studies delivered the intervention exclusively by group sessions^33^, ^37^, ^38^ and three by individual sessions,^28^, ^35^, ^36^ whilst six utilized a mixture of both. All studies began with frequent contacts, be it weekly or monthly, and stepped this down to bi‐weekly, monthly, and bi‐monthly as time went on. All the studies utilized registered health professionals, dieticians, or nutritionists, to lead the interventions.

As healthy eating and physical activity are considered standard health advice, all the dietary intervention groups received basic advice on exercise. All the control groups received in‐person general verbal and written advice relating to diet and exercise but no specific individualized advice, this was considered as none, standard or minimal intervention.

### 3.5 Risk of bias

As is the nature of ambulatory dietary interventions all studies were at high risk of performance bias due to lack of blinding of participants and personnel to the intervention. The risk of bias tool was modified to consider blinding of weight outcome assessment and inflammatory markers separately (Figure S3). The former was high risk in four studies and the latter low risk in two‐thirds. Visual inspection off a funnel plot shows asymmetry suggestive of a small study bias for weight (Figure S4).

## 4 OUTCOMES

The outcome values for weight, IL‐6, and TNF‐α, reported at trial end (range 12 to 36 months) were analyzed.

### 4.1 Change in weight

The mean differences in weight (kg) between intervention and control groups were comparatively similar for the three subgroups analyzed; diet versus control, diet and exercise versus exercise, and diet and exercise versus control, at −5.38, −5.52, and −5.52 kg, respectively. Overall, participants randomized to receive a dietary weight loss intervention achieved greater reduction in weight compared to control MD = −5.41 kg (95% CI ‐7.14, −3.68) (Figure S5). Prespecified sensitivity analysis assessed the influence of blinding of weight loss outcome assessment. Including studies at high risk of this bias had attenuated the degree of weight loss rather than overestimating it (Figure S6). Mean weight change for the intervention arms ranged from −1.14 kg to −14 kg, and within the control groups between −4.1 kg to + 0.3 kg, equating to changes from −1.6% to −15%, and −4.4% to +0.5%, respectively. Weight loss reached reported statistical between‐group significance in all but one of the comparison groups (Table S2).

### 4.2 Serum IL‐6

Due to the skewed distribution of IL‐6 a meaningful analysis of pooled data was not possible. All weight loss arms reported a change in serum IL‐6 ranging from −33% to −7% at final follow‐up, whilst change in the control groups ranged from −19% to a 33% increase. Reported between‐arm differences were statistically significant in nine of the comparisons encompassing serum IL‐6 reductions equally represented across the three different comparisons.

### 4.3 Serum TNF‐α

Three studies including 629 participants analyzed serum TNF‐α between‐group differences at final follow‐up, none of which reported statistical significance.^30^, ^31^, ^33^ Again, heterogeneity in measures of central tendency meant valid pooled analysis of data was not possible. Changes in TNF‐α ranged from −21% to +25% in the weight loss groups and −20% to +8% in controls.

### 4.4 Comparisons of changes in weight, serum IL‐6 and TNF‐α

Table 1 presents studies in descending order of percentage weight loss in relation to changes in IL‐6 and TNF‐α. Ten of the intervention arms achieved between ≥5% and 15% weight loss^29^, ^30^, ^31^, ^32^, ^34^, ^37^, ^38^, ^39^ with four reporting <5% loss.^33^, ^35^, ^36^

**TABLE 1:** The effect of dietary weight loss intervention on percentage change serum inflammatory markers by descending weight loss (%).

	Comparison arms, n=	Mean age (SD), % female	Participant characteristic	Baseline weight kg (SD)	Intervention	% weight loss within group (between group p value)	Within group % change in IL‐6 (between group p value)	Within group % change TNF (between group p value)	
**Esposito** ^37^ **2004**	*Diet & exercise n = 55* *Control n = 55*	43.5 (4.8), 0 43 (5.1), 0	Erectile dysfunction	103 (9.4) 101 (9.7)	CR Mediterranean diet 24 months	−15 −2 P =0.007	−31 +2 P =0.03	NA	≥ **10% weight loss**
**Esposito** ^38^ **2003**	*Diet & exercise n = 60* *Control n = 60*	34.2, 100 35, 100	Pre‐menopausal	95 (9.4) 94 (9.2)	CR Mediterranean diet 24 months	−15 −3 P < 0.001	−33 −7 P =0.009	NA	
**Nicklas** ^30^ **2004: a**	*Diet n = 53* *Control n = 60*	68 (5), 74 69 (6), 66	OA	95.6 (152) 95.7 (18.8)	CR diet 18 months	−13 −2.4 P < 0.0001	−15 +6 P =0.009	+26 −21 P =0.67	
**Messier** ^32^ **2013**	*Diet & exercise n = 152* *Exercise n = 150*	65 (6), 72 66 (6), 72	OA	93 (14.4) 92 (14.5)	CR diet 18 months	−11.4 −2 P < 0.001	−15 0 P =0.007	NA	
**Imayama** ^34^ **2012: b**	*Diet & exercise n = 117* *Exercise n = 117*	58.0 (4.5), 100 58.1 (5.0), 100	Postmenopausal	82.5 (10.8) 83.7(12.3)	CR diet 12 months	−10.8 −2.4 P < 0.0001	−16 +7 P < 0.001	NA	
**Nicklas** ^30^ **2004: b**	*Diet & exercise n = 53* *Exercise n = 53*	68 (7), 74 69 (6), 74	OA	91.8 (17.4) 92.4 (14.6)	CR diet 18 months	−9 −4.4 NA	−7 +0.5 NA	−21 +8 NA	≥**5 to <10% weight loss**
**Imayama** ^34^ **2012: a**	*Diet n = 118* *Control n = 87*	58.1 (6.0), 100 57.4 (4.4), 100	Postmenopausal	84 (11.8) 84.2 (12.5)	CR diet 12 months	−8.5 −0.8 P < 0.0001	−14 +12 P =0.001	NA	
**Beavers** ^29^ **2013**	*Diet & exercise n = 98* *Exercise n = 97*	66.8 (4.6), 67 67.2 (4.8), 64	Recent CVD incident or metabolic syndrome diagnosis	92.8 (16.1) 91.7 (13.1)	CR diet 18 months	−7.7 −1 P < 0.001	−14 +4 P < 0.05	NA	
**Miller** ^31^ **2014** ^**1**^	*Diet & exercise n = 151* *Control n = 150*	60.3 (9.8), 70 54.9 (7.2), 70	IGT	94.4 (14.7) 93 (16.2)	CR diet 12 months	−7.8 −1.7 P < 0.001	−33 +33 P =0.001	−6 −17 P =0.083	
**Herder** ^39^ **2009**	*Diet & exercise n = 207* *Control n = 199*	55.7 (7.1), 64 55.1 (6.9), 66	IGT	86.9 (14.2) 86.0 (14.7)	CR diet 12 months	−5.3 −1.4 P < 0.001	−17 +0.6 P =0.033	NA	
**Ard** ^33^ **2017**	*Diet & exercise n = 55* *Exercise n = 54*	70.3 (4.8), 48 69.9 (4.5), 69	≥ 1 medication for hyperlipidaemia, HTN or diabetes	94.1 (2.1)* 95.2 (1.7)*	CR diet 12 months	−4 −1.4 P < 0.01	−13 +3 P =0.111	−2 +9 P =0.048	<5% weight loss
**Welsh** ^36^ **2016**	*Diet & exercise n = 75* *Control n = 76*	52.6 (10.3), 66 52.4 (9.8), 55	Pakistani or Indian ethnic origin, IGT	79.2 (16.7) 80.5 (15.5)	CR diet 36 months	−1.4 +0.4 P =0.026	−21 −19 P =0.899	NA	
**Thompson** ^35^ **2014**	*Diet n = 248* *Control n = 99*	60 (10), 36 60 (11), 37	Newly diagnosed T2DM	90.2 (16.7) 93.9 (19.0)	CR diet 12 months	−1.6 −0.4 P < 0.0001	−8 −11 NA	NA	
**Serra‐Prat** ^28^ **2022**	*Diet & exercise n = 77* *Control n = 95*	69.6 (2.7), 69 69.9 (2.7), 63	Features of metabolic syndrome and/or obesity‐related hypoventilation syndromes	Not stated	CR diet 24 months	−2.7** −1.7** P > 0.05	−27 +24 p = 0.221	NA	

**CR**: Calorie restriction, **OA**: Osteoarthritis, **T2DM**: Type II Diabetes mellitus, **IGT**: Impaired glucose tolerance, **CVD**: cardiovascular disease, **HTN**: Hypertension, **IL‐6**: Interleukin 6, **TNF‐α**: Tumor necrosis factor.

**NA**: Not available.

* **SE**.

** Mean Kg (percentage change not available).

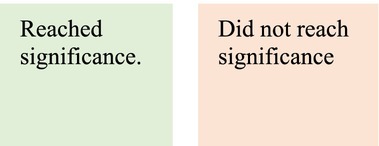

^1^Subsample n = 15 of HELP PD study^40^.

A table including all studies' outcomes during and/or on completion of the trial can be found in Table S3. Those that reported outcomes at more than one‐time point are displayed in Table 2. Of those studies reporting the change in weight throughout an intervention phase only, one showed continued weight loss,^32^ one maintenance,^33^ and the third a small gain,^35^ though all remained statistically significant throughout. Of the three trials including a follow‐on maintenance phase, weight loss continued in the diet group in Nicklas et al,^30^ whilst very slightly attenuating but remaining significant in Beavers et al^29^ Serra‐Pratt et al reported significant weight loss at the end of a 6‐month intervention which was maintained at 12 months but partially regained by 24 months.^28^

**TABLE 2:**
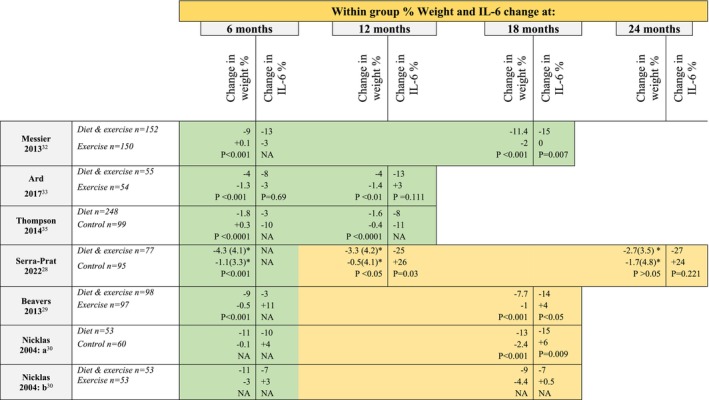
Percentage change in weight and IL‐6 over time.

NA: Not Available.

* Mean Kg (SD) (percentage change not available).

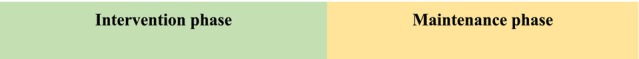

## 5 DISCUSSION AND INTERPRETATION OF RESULTS

This systematic review focused on randomized controlled trials that investigated the effect of dietary weight loss interventions on the inflammatory markers IL‐6 and TNF‐α in participants with obesity with at least 12 months of follow‐up.

The mean difference in weight (kg) between intervention and control groups was comparatively similar for the three subgroups analyzed; diet versus control, diet and exercise versus exercise, and diet and exercise versus control. This is congruent with the similarity in both degrees of calorie restriction and macronutrient content across the interventions. In addition, all groups received some exercise advice. Previous meta‐analysis has reported that exercise alone does not significantly reduce weight or inflammatory markers.^41^

Considering the percentage of weight loss in relation to changes in IL‐6, those that achieved between ≥5% and 15% weight loss were all accompanied by reported statistically significant decreases in serum IL‐6 compared to controls.^29^, ^30^, ^31^, ^32^, ^34^, ^37^, ^38^, ^39^ In contrast there was no significant reduction in serum IL‐6 in the three trials reporting <5% weight loss.^33^, ^35^, ^36^ Baseline weight was not provided in one study therefore percentage weight loss was not calculated. However, mean weight loss at 24 months was −2.7 kg (3.5) (reported p > 0.05), which in this cohort is unlikely to represent greater than 5% weight loss, and in keeping IL‐6 was not significantly reduced (reported p = 0.221).^28^ Consistent with this finding, Imayama et al further analyzed their dietary intervention arm stratified by participants that achieved ≥5% and <5% weight loss, reporting reductions in IL‐6 of 29.9% and 7.7%, reported p < 0.001 and p = 0.429, respectively, compared to control.^34^

As only three studies provided TNF‐α data no pattern between weight loss and serum TNF‐α was observable.

A 2023 meta‐analysis of RCTs performed subgroup analysis of five, 24‐week or less, calorie restriction weight loss trials, reporting no significant difference in serum TNF‐α concentrations between the intervention and control groups (reported SMD 0.22 pg/ml [95% CI 0.11 to 0.55], p = 0.19).^42^

TNF‐α has a short half‐life and wide population variability making it less desirable as a marker to monitor inflammation.^43^ Alternatively, circulating TNF‐α soluble receptors (sTNFR) are suggested to be a more stable reflection of long‐term TNF‐α activity.^44^ Two of the included studies measured sTNFR. Beavers et al found lower but non‐significant post‐intervention sTNFR‐1 levels in the diet and physical activity group, 1344 pg/ml (95% CI 1296–1393) versus control 1426 pg/ml (95% CI 1372–1482), reported p = 0.09 (TNF‐α was not measured).^29^ Whilst Nicklas et al reported dietary weight loss resulted in a significant reduction in sTNFR‐1 compared to no weight loss (change in log sTNFR1 = −0.070 SD 0.017 compared to −0.013 SD 0.017 pg/ml; P = 0.007), but no effect on either sTNFR‐2, or TNF‐α.^30^

A few trials independently measured a range of circulating biomarkers, spanning cell adhesion molecules, fibrinolytic enzymes, hepatocyte acute phase proteins, e.g., serum amyloid‐A, as well as the adipokine hormones leptin and adiponectin.

Prospective population and cross‐sectional studies consistently report increased incidence and risk of disease for those in the highest percentiles of serum inflammatory markers compared to the lowest. However, what constitutes a normal range of IL‐6 and TNF‐α, or a threshold considered a risk factor for, or marker of disease is unclear.^45^ Statistically significant changes in IL‐6 cannot necessarily be interpreted as clinically significant. Only one study was powered for changes in IL‐6, with Messier et al using a 20% difference in group means based on their own preliminary data.^32^

A longitudinal study of over 65‐year‐olds compared plasma IL‐6 in the baseline samples of participants who had developed mobility‐disability four years later to those that had not, reporting a non‐linear increase in risk rising rapidly once circulating IL‐6 became greater than 2.5 pg/ml.^46^ Further analysis of their data by Messier et al showed that the odds of achieving an IL‐6 less than 2.5 pg/ml more than doubled once weight loss reached 5%.^32^ However, half of the studies in this review had baseline IL‐6 greater than 2.5 pg/ml, spanning all ages, health states, and pathology, none of which achieved IL‐6 levels less than 2.5 pg/ml despite weight loss ranging from 7.7% to 15%.

This systematic review selected studies with at least 12 months of data to examine the length of intervention required to reduce inflammatory markers and the effect of weight stability and regain over time, so that results may be meaningful for realistic clinical practice. In the two‐year study by Serra‐Prat et al weight loss achieved during the 6 month intervention and maintained at 12 months corresponded with significant reductions in IL‐6, however, both lost significance with weight regain at 24 months.^28^ Trials that did achieve and maintained ≥5% weight loss show varied patterns of change in IL‐6, however all remained significantly reduced throughout. Messier et al reported that as participants weight progressively fell from −9% to −11% at 6 and 18 months of intervention so did IL‐6 from −12.5% to −15%, respectively.^32^ Similarly, Nicklas et al reported further reductions in IL‐6 in the diet‐only arm as they continued to lose weight, whereas weight loss began to attenuate in the diet and exercise arm between 6 and 18 months but IL‐6 reductions stayed stable.^30^ Beavers et al also reported a slight weight increase during a 12 months maintenance phase but in contrast, IL‐6 continued to decrease.^29^ In the two trials in which weight loss was <5% throughout IL‐6 did not significantly reduce at any time.^33^, ^35^ Both trials by Esposito et al lasted 2 years and achieved 15% weight loss with significant reductions in IL‐6.^37^, ^38^ Although measurements were not taken throughout this does suggest that the effect of maintained weight loss on inflammatory markers is not short‐lived and maintained over time.

Overall interpretation of the small number of studies reporting change over time suggests that if ≥5% weight loss is achieved and maintained IL‐6 is likely to remain significantly reduced. That a significant reduction in IL‐6 remained despite some attenuation of weight loss over time suggests that once the weight loss is achieved marked calorie restriction may no longer be required. However, it remains unclear if the initial reduction is driven by weight loss or calorie restriction. Further analysis in two of the studies reported that changes in IL‐6 related to changes in weight and/or BMI,^38^, ^39^ whilst Nicklas et al reported that reductions in IL‐6 in the dietary intervention groups were not necessarily related to weight loss.^30^ Decades of animal studies have reported downregulation of inflammatory cytokine production in response to caloric restriction.^47^ However, it is not clear in humans if this is dependent on dose (degree of calorie restriction) or baseline weight. As all studies included in this review had a similar calorie intake, with none prescribing a very low‐calorie diet, further interpretation cannot be made. Overall, previous studies comparing macronutrient content, glycemic load, specific food type, and time‐restricted eating have not found significant between‐group differences, with none appearing to effect serum inflammatory markers over or above energy restriction and/or weight loss.^48^, ^49^, ^50^, ^51^, ^52^, ^53^ Irrespective of the mode of action, that dietary weight loss intervention remains efficacious in IL‐6 reduction with weight maintenance is potentially clinically relevant to disease management and prevention.

Previous studies have reported that circulating inflammatory cytokines are higher in older adults and post‐menopausal women.^20^, ^23^ This systematic review included five studies with mean age of participants over 60 years.^28^, ^29^, ^30^, ^32^, ^33^ Within these studies the degree of weight loss still remained the most important factor, with the three achieving ≥5% weight loss showing significant reductions in IL‐6, and the two with <5% no effect. The same applies to the one study of post‐menopausal women who achieved ≥5% weight loss with a corresponding significant reduction in IL‐6.^34^ However, this pattern of results may be influenced by potential publication bias as evidenced in the left skewed funnel plot (Figure S4).

There were no apparent differences in outcomes between the sexes. Serra‐Prat et al. reported weight loss by sex, with similar significant mean loss at 6, 12, and regain by 24 months in both men and women.^28^ However, IL‐6 levels were reported as a whole cohort so any potential sex differences in IL‐6 for comparable weight loss cannot be determined. Esposito et al conducted two studies with the same methodology, one in men with erectile dysfunction^37^ and the other pre‐menopausal women.^38^ Both intervention groups lost 15% of their body weight, with corresponding reductions in IL‐6 of −31% (reported p = 0.03) in men and −33% (reported p = 0.009) in women.

Participants had a range of pathology spanning both metabolic and non‐metabolic diseases. The wider literature suggests that obesity phenotype and body fat distribution play a role in associated disease expression, potentially through regional variations in WAT adipokine expression.^54^ However, the heterogeneity of included pathology in this review did not appear to affect the results, again supporting that it is the degree and maintenance of successful weight loss is the most important factor.

To reduce the risk of, and/or improve pre‐existing obesity co‐morbidities, expert consensus and guidelines recommend targeting a weight loss of 5% or more (commonly described as ~ 5 kg in those with BMI 25–35 kg/m^2^) which has been shown to improve outcomes across a spectrum of disease.^40^ Our findings are in keeping with this, as significant IL‐6 reductions occurred only with ≥5% weight loss. In the largest study, Herder et al (n = 406), reported a 12‐month intervention outcome of −5.3% weight loss (−4.63 Kg − 5.29, −3.96) compared to control, −1.4% (−1.26 Kg − 1.76,‐0.76) which equated to mean BMI reductions of −1.67 kg/m^2^ (−1.9, −1.42) and −0.46 kg/m^2^ (−0.64, −0.28) reported p < 0.001, respectively.^39^ Current guidelines advocate focusing on reducing risk, the effects of co‐morbidities, and increasing wellbeing over specific weight or BMI targets.^55^

A weight loss of 5% is a challenge for individuals in the current obesogenic environment. However, pragmatic trials such as STANDby and DiRECT have shown this can be achieved, although maintenance remains a challenge.^56^, ^57^

## 6 STRENGTHS AND LIMITATIONS

This systematic review included only long‐term randomized controlled trials of the effect of dietary interventions on markers of inflammation. Strengths of this review include the long follow‐up time and only including comparable interventions. The review includes studies across all adult age groups involving only men, only women, and various percentages of both, with and without underlying pathology, increasing the generalizability of results.

However, none of the trials explicitly excluded those with auto‐immune disease and only one mentioned excluding people taking long‐term corticosteroids, which could influence circulatory levels of markers assessed here.^36^ As with most ambulatory dietary interventions, blinding of participants and some investigators is not possible and as a result, this may have influenced the quality of the studies. All of the studies were conducted in western countries, and, with the exception of one Scottish study in which South East Asian origin was an inclusion criterion, at least three‐quarters of participants were white and the results may not be applicable to other countries. BMI was necessarily used as a means to define and identify obesity but may not be the best measurement of excess adiposity.

## 7 CONCLUSION

Pathological expansion of adipose tissue with alteration in adipokine secretion is considered one underlying mechanism of obesity‐driven disease. Overall, this systematic review suggests that weight loss reduces circulating IL‐6 in people with obesity across all life stages and a range of diseases. To have a significant lasting effect the reduction in body weight required appears likely to be at least 5% in the long term. The effects of weight loss on circulating TNF‐α remain inconclusive.

Embedding weight loss interventions into both primary and secondary care to reduce underlying inflammatory load should be an integral part of healthcare in both disease prevention and treatment. Studies quantifying serum inflammatory markers with validated clinical endpoints and/or disease risk stratification may support this.

## CONFLICT OF INTEREST

Cate Bulmer‐ none declared. Alison Avenell – none declared.

## Supporting information


**Figure S1:** Search strategy and results for MEDLINE (Ovid) and Embase (Ovid).
**Figure S2**: A Modified PRISMA Flow Diagram.
**Table S1**: Characteristics of Included studies.
**Figure S3**: Quality assessment of all RCT's.
**Figure S4**: Funnel plot: Change in weight subgroups.
**Figure S5**: Forest plot.
**Figure S6**: Forest plot sensitivity analysis‐ Blinding of weight loss outcome.
**Table S2**: Effect of dietary weight loss intervention studies on weight loss (kg) and serum inflammatory markers (%).
**Table S3**. Effect of dietary weight loss intervention on percentage change weight and serum inflammatory markers over time.
