# Supplementary material for: The effect of dietary weight‐loss interventions on the inflammatory markers interleukin‐6 and TNF‐alpha in adults with obesity: A systematic review and meta‐analysis of randomized controlled clinical trials
Source: Obes Rev. 2025 Mar 16;26(7):e13910. doi: 10.1111/obr.13910 (PMC12137039; doi:10.1111/obr.13910)
Supplement: Supplementary file 1 — Figure S1: Search strategy and results for MEDLINE (Ovid) and Embase (Ovid). Figure S2: A Modified PRISMA Flow Diagram. Table S1: Characteristics of Included studies. Figure S3: Quality assessment of all RCT's. Figure S4: Funnel plot: Change in weight subgroups. Figure S5: Forest plot. Figure S6: Forest plot sensitivity analysis‐ Blinding of weight loss outcome. Table S2: Effect of dietary weight loss intervention studies on weight loss (kg) and serum inflammatory markers (%). Table S3. Effect of dietary weight loss intervention on percentage change weight and serum inflammatory markers over time. [file OBR-26-e13910-s001.pdf]

The Effect of Dietary Weight-loss Interventions on the Inflammatory markers Interleukin-6 and TNF-alpha in adults with Obesity: A Systematic Review and Meta-Analysis of Randomised Controlled Clinical Trials

Authors:

Cate Bulmer*, Health Services Research Unit, University of Aberdeen, UK

Alison Avenell, Health Services Research Unit, University of Aberdeen, UK

***Corresponding author:**

Dr Cate Bulmer

Health Services Research Unit, 3rd Floor, Health Sciences Building, Foresterhill, AB25 2ZD Aberdeen

Email: catherine.bulmer@nhs.scot

Supplementary material

Figure S1: Search strategy and results for MEDLINE (Ovid) and Embase (Ovid)

Database(s): **Ovid MEDLINE(R) ALL** 1946 to November 19, 2024

Search Strategy:

#	Searches	Results
1	exp Obesity, Morbid/ or exp Obesity/ or exp Obesity Hypoventilation Syndrome/ or obesity.mp. or exp Obesity, Abdominal/ or exp Obesity, Metabolically Benign/	440908
2	obes$.mp.	478610
3	Overweight/	36250
4	overweight.mp.	101489
5	excess weight.mp.	7089
6	exp Weight Loss/	52545
7	(weight adj2 (los$ or reduc$)).mp.	163422
8	(reduc$ adj2 (bmi or body mass index)).mp.	4343
9	weight loss.mp.	131843
10	(reduction adj2 ("visceral fat" or "ectopic fat")).mp.	200
11	(reduction adj2 ("visceral adipose" or "ectopic adipose")).mp.	66
12	Interleukin-6.mp. or exp Interleukin-6/	116212
13	IL 6.mp.	165173
14	TNFalpha.mp. or exp Tumor Necrosis Factor-alpha/	161427
15	TNFalfa.mp.	17
16	tumo?r necrosis factor al???.mp.	202137
17	Tumor Necrosis Factor?.mp.	239213
18	(Adipokines and Interleukin 6).mp.	1228
19	(adipokines and IL 6).mp.	1489
20	(Adipokines and TNF alpha).mp.	1234
21	(Adipokines and tumo?r necrosis factor al???).mp.	1398
22	(adipocytokines and interleukin-6).mp.	335
23	(adipocytokines and TNF-alpha).mp.	381
24	randomi?ed controlled trial.pt.	626497
25	controlled clinical trial.pt.	95641
26	randomized.ab.	669526
27	placebo.ab.	253854
28	drug therapy.fs.	2755552
29	randomly.ab.	447003
30	trial.ab.	725245
31	groups.ab.	2767015
32	exp bariatric surgery/ or exp gastric bypass/ or exp gastroplasty/ or exp jejunoileal bypass/ or exp lipectomy/	36098
33	pregnan$.ti.	280668
34	breast feeding.mp. or exp Breast Feeding/	51934
35	exp adolescent/ or exp child/ or exp infant/	4110532
36	exp animals/ not humans.sh.	5278797
37	exp HIV/ or exp HIV Infections/ or HIV.mp.	462090
38	cancer.mp. or exp Neoplasms/	4738261
39	or/1-11	606945
40	or/12-23	388927
41	or/24-31	6128958
42	or/32-38	13930249
43	39 and 40 and 41	6552
44	43 not 42	2976

Database(s): **Embase** 1974 to 2024 November 20

Search Strategy:

#	Searches	Results
1	exp *obese patient/ or exp *obesity/ or exp *body weight disorder/ or exp overnutrition/ or exp *abdominal obesity/ or exp *diabetic obesity/ or exp *lipedema/ or exp *metabolic syndrome x/ or exp *metabolically benign obesity/ or exp *morbid obesity/ or exp *normal weight obesity/ or exp *obesity associated inflammation/ or exp *obesity hypoventilation syndrome/ or exp *obesity related glomerulopathy/	758820
2	obes$.mp.	805790
3	Overweight/	353637
4	overweight.mp.	146515
5	excess weight.mp.	11885
6	exp body weight loss/ or exp body weight change/ or exp body weight management/	228699
7	(weight adj2 (los$ or reduc$)).mp.	337555
8	(reduc$ adj2 (bmi or body mass index)).mp.	7333
9	weight loss.mp.	239714
10	(reduction adj2 ("visceral fat" or "ectopic fat")).mp.	346
11	(reduction adj2 ("visceral adipose" or "ectopic adipose")).mp.	98
12	Interleukin-6.mp. or exp Interleukin-6/	387424
13	IL 6.mp.	237661
14	TNFalpha.mp. or exp Tumor Necrosis Factor-alpha/	309774
15	TNFalfa.mp.	153
16	tumo?r necrosis factor al???.mp.	264234
17	Tumor Necrosis Factor?.mp.	565444
18	(Adipokines and Interleukin 6).mp.	3302
19	(adipokines and IL 6).mp.	1946
20	(Adipokines and TNF alpha).mp.	1430
21	(Adipokines and tumo?r necrosis factor al???).mp.	1895
22	(adipocytokines and interleukin-6).mp.	981
23	(adipocytokines and TNF-alpha).mp.	566
24	exp clinical trial/ or clinical trial.mp.	2390252
25	controlled clinical trial.mp.	506643
26	randomized.ab.	973320
27	placebo.ab.	376428
28	drug therapy.fs.	4681261
29	randomly.ab.	594837
30	trial.ab.	1069340
31	groups.ab.	3874684
32	exp bariatric surgery/ or exp gastrointestinal surgery/ or exp biliopancreatic bypass/ or exp gastric banding/ or exp sleeve gastrectomy/ or exp bariatrics/	451780
33	pregnan$.ti.	324892
34	breast feeding.mp. or exp Breast Feeding/	77353
35	exp adolescent/ or exp child/ or exp infant/	4189333
36	exp animal/ not exp human/	5344603
37	exp conference paper/ or exp conference abstract/ or exp "conference review"/	3225449
38	exp Human immunodeficiency virus/ or exp Human immunodeficiency virus infection/ or HIV.mp.	939944
39	exp malignant neoplasm/	4539877
40	or/1-11	1188436
41	or/12-23	775577
42	or/24-31	9788043
43	or/32-39	16562591
44	40 and 41 and 42	22653
45	44 not 43	9583
46	limit 45 to exclude medline journals	1539

Figure S2: A Modified PRISMA Flow Diagram

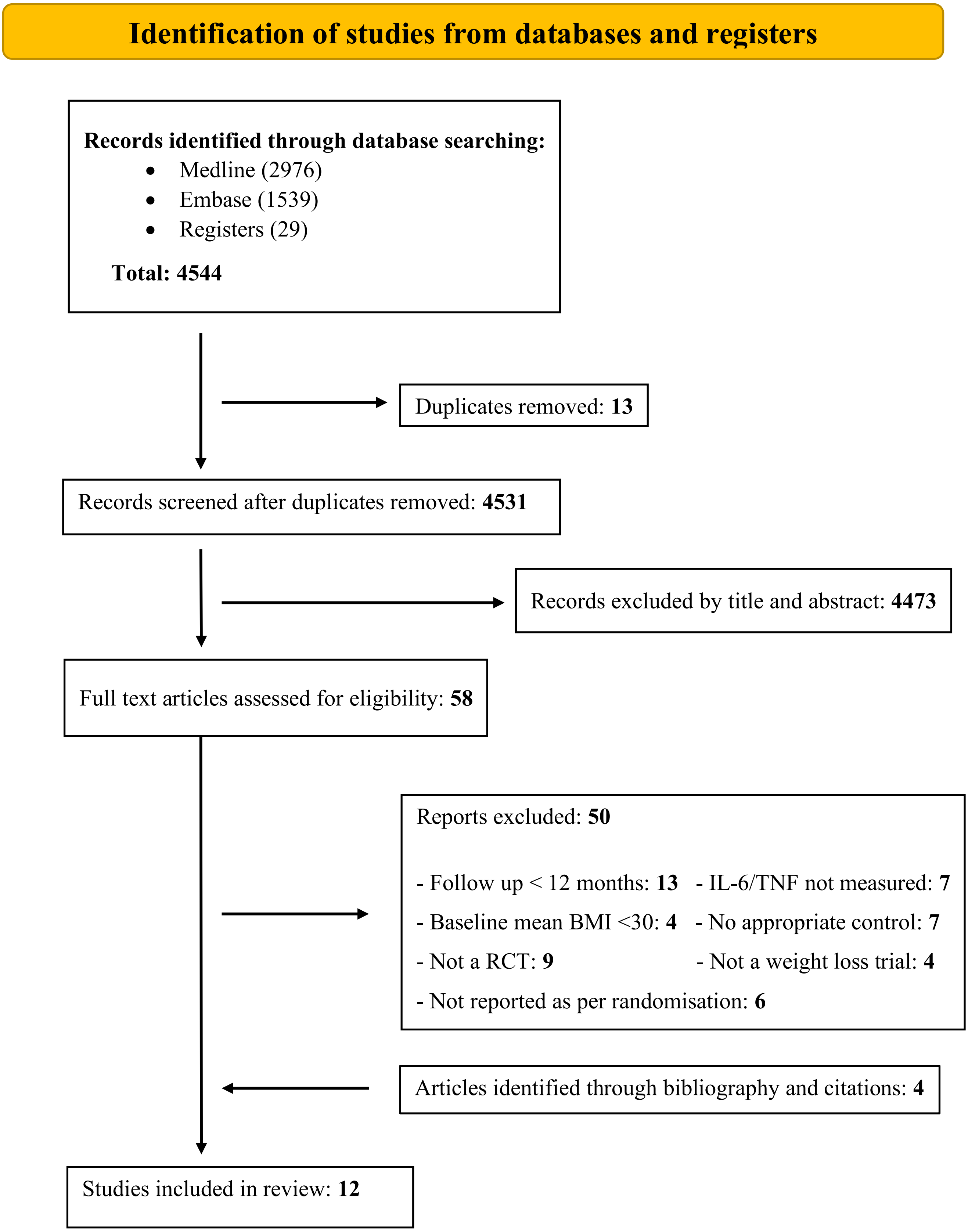

Table S1: Characteristics of Included studies

Author, Year and Country	Study characteristics	Participants	Intervention	Primary study outcome
**Esposito 2003, Italy**[38]	**Inclusion criteria:**<br>Female, BMI ≥30, pre-menopausal, age 20 to 46 years<br><br>**Exclusion criteria:**<br>T2DM, IGT, HTN, CVD, psychiatric problems, history of alcohol abuse, current smoker, any medication use, pregnancy.	**Participants (n)**<br>Diet & exercise: 60<br>Control: 60<br>**Sex:**<br>100% female<br>**Mean age (SD) at baseline, years:**<br>Diet & exercise: 34.2<br>Control: 35.0<br>**Mean weight (SD) at baseline, kg:**<br>Diet & exercise: 95 (9.4)<br>Control: 94 (9.2)<br>**Mean BMI (SD) at baseline:**<br>Diet & exercise: 35 (2.3)<br>Control: 34.7 (2.4)<br><br>**IL-6 pg/ml baseline median (IR):**<br>Diet & exercise: 4.3 (1.9-9.0)<br>Control: 4.1 (2.0-9.0)<br>**TNF pg/ml baseline:**<br>Diet & exercise: NA<br>Control: NA	**Arms:** Diet & exercise v's Control<br>**Duration:** 24 months<br>**Intervention:**<br>***Dietary advice and delivery:***<br>Individualised nutritionist advice to loose 10% total body weight. Dietary advice was tailored to each woman. Delivered via small group sessions monthly during the first year and bimonthly during the second year. Behavioural and psychological counselling was also offered.<br>***Recommended macronutrient content:***<br>Mediterranean-style diet with 50% to 60% carbohydrates, 15% to 20% proteins, less than 30% total fat, less than 10% saturated fat, 10% to 15% monounsaturated fat, 5% to 8%polyunsaturated fat, and 18 g of fiber per 1000 kcal.<br>***Calories:***<br>The mean caloric intake goal was set at 1300 kcal/d for the first year and 1500 kcal/d for the second year.<br>***Exercise:***<br>Individual guidance on increasing level of physical activity through monthly sessions with an exercise trainer.<br><br>**Control:**<br>Given general oral and written information about healthy food choices and exercise at monthly visits, no specific individualised programs were offered.	**Primary outcome:**<br>Lipid and glucose intake; blood pressure; homeostatic model assessment of insulin sensitivity; and circulating levels of interleukin 6, interleukin 18, C-reactive protein , and adiponectin
**Esposito 2004, Italy**[37]	**Inclusion criteria:**<br>Men with erectile dysfunction, BMI ≥30, age 35-55,	**Participants (n)**<br>Diet & exercise: 55<br>Control: 55<br>**Sex:**	**Arms:** Diet & Exercise v's Control<br>**Duration:** 24 months<br>**Intervention:**<br>***Dietary advice and delivery:***	**Primary outcomes:**<br>Erectile function score, levels of

	**Exclusion criteria:**<br>Recent diet reduction programme, DM/IGT, CKD, HTN, CVD, pelvic trauma, prostatic disease, peripheral or autonomic neuropathy, psychiatric problems, drug/alcohol abuse.	100% male<br>**Mean age (SD) at baseline, years:**<br>Diet & exercise: 43.5 (4.8)<br>Control: 43 (5.1)<br>**Mean weight (SD) at baseline, kg:**<br>Diet & exercise: 103 (9.4)<br>Control: 101 (9.7)<br>**Mean BMI (SD) at baseline:**<br>Diet & exercise: 36.9 (2.5)<br>Control: 36.4 (2.3)<br>**IL-6 pg/ml baseline median (IR):**<br>Diet & exercise: 4.5 (1.9-9.0)<br>Control: 4.4 (2.0 – 9.0)<br>**TNF pg/ml baseline median (IQR):**<br>Diet & exercise: NA<br>Control: NA	Individualised nutritionist advice to loose 10% total body weight over 2 years. Dietary advice was tailored to each man Delivered via small group sessions monthly during the first year and bimonthly sessions during the second year. Behavioural and psychological counselling was also offered.<br>***Recommended macronutrient content:***<br>carbohydrates, 50% to 60%; proteins,15%to 20%; total fat, less than 30%;saturated fat, less than10%;mono- unsaturated fat, 10% to 15%; polyunsaturated fat, 5% to 8%; and fiber, 18 g.<br>***Calories:***<br>The mean daily caloric intake was 1700 kcal for the first year and 1900 kcal for the second year.<br>***Exercise:***<br>Individual guidance on increasing level of physical activity through monthly sessions with an exercise trainer.<br><br>**Control:**<br>General oral and written information about healthy food choices and exercise at baseline and at subsequent bi-monthly visits, but no specific individualised program was provided.	cholesterol and triglycerides, interleukin 6, interleukin 8, and C-reactive protein, and endothelial function as assessed by vascular responses to L-arginine
**Nicklas 2004, USA**[30]	**Inclusion criteria:**<br>Aged ≥ 60 years, BMI ≥ 28, sedentary, knee pain, radiographic evidence of knee OA<br>**Exclusion criteria:**<br>No radiographic evidence of tibiofemoral OA, contraindications for participation in an exercise program; severe HTN, recent stroke, COPD, T1DM, psychiatric disease, renal disease, liver disease, active cancer other than skin cancer, or	**Participants (n)**<br>Diet only: 71<br>Diet + exercise: 64<br>Exercise:67<br>Control: 70<br>**Sex (% female):**<br>Diet only: 74<br>Diet + exercise: 74<br>Exercise:74<br>Control arm: 66<br>**Mean age (SD) at baseline, years:**<br>Diet only: 68 (5)<br>Diet + exercise: 68 (7)	**Arms:** Diet v's Control, Diet & exercise v's Exercise<br>**Duration:**18 months<br>**Interventions:**<br>***Dietary advice and delivery:***<br>To produce and maintain an average weight loss of 5% of baseline body weight. Three phases: Intensive months 1–4; one introductory individual session was followed by 16 sessions (3 group sessions and 1 individual session per month). Transition: months 5–6; bi-weekly contacts. Maintenance phase: months 7–18, monthly meeting and phone 2 weekly.<br>***Recommended macronutrient content:***<br>Low fat diet<br>***Calories:***<br>Decrease energy intake by 500 kcal/d, aiming for a loss of ~0.5 kg body weight/week	**Primary outcome:** Self-reported physical function and disability – (ADAPT study)<br><br>**Ancillary study** serum inflammatory biomarkers

	anaemia; cognitive impairment or consumed ≥14 alcoholic drinks/week	Exercise:69 (6)<br>Control arm: 69 (6)<br>**Mean weight (SD) at baseline, kg:**<br>Diet only: 95.6 (15,2)<br>Diet + exercise: 91.8 (17.4)<br>Exercise: 92.4 (14.6)<br>Control arm: 95.7 (18.8)<br>**Mean BMI (SD) at baseline:**<br>Diet only: 34.4 (4.9)<br>Diet + exercise: 33.9(5.6) Exercise:34.6 (5.8)<br>Control arm: 34.5 (5.3)<br>**IL-6 pg/ml baseline mean (SD):**<br>Diet only: 4.7 (3.4)<br>Diet + exercise: 4.9 (3)<br>Exercise: 4.4 (3.1)<br>Control arm: 4.7 (3.2)<br>**TNF pg/ml baseline mean (SD):**<br>Diet only: 2.5 (1.8)<br>Diet + exercise: 3.4 (6.4)<br>Exercise:<br>Control arm: 3.8 (7.5)	***Exercise:***<br>45-minute exercise program three days per week consisting of aerobic and resistance-training. The first 4 months facility-based after which participants who wished to exercise at home underwent a 2-month transition phase in which they alternated between the facility and the home<br><br>**Control:**<br>Group sessions providing attention, social interaction, and general health education. Monthly for 3 months and discussed topics concerning osteoarthritis, obesity, and exercise. Monthly phone contact was maintained during months 4–6 and bimonthly contact was maintained during months 7–18.	
**Herder 2009, Finland**[39]	**Inclusion criteria:**<br>Aged 40–65 years, overweight or obese (BMI≥25 kg/m2) and IGT.<br><br>**Exclusion criteria:**<br>Diabetes mellitus, hypoglycaemic medications, regular vigorous exercise pro- gramme, any chronic disease making a 6-year survival improbable, clinical	**Participants (n)**<br>Diet and exercise: 207<br>Control arm: 199<br>**Sex (% female):**<br>Diet and exercise: 64%<br>Control: 66%<br>**Mean age (SD) at baseline, years:**<br>Intervention arm: 55.7 (7.1)<br>Control: 55.1 (6.9)<br>**Mean weight (SD) at baseline, kg:**<br>Diet and exercise: 86.9 (14.2)	**Arms:** Diet and exercise v's Control<br>**Duration:** 12 months<br>**Intervention:**<br>***Dietary advice and delivery:***<br>Nutritionist face-to-face dietary advice tailored to each subject. Individual and group sessions at 1 to 2 weeks, 5 to 6 weeks, 3, 4 and 6 months and thereafter every 3 months. Goal BMI <25<br><br>***Recommended macronutrient content:***<br>Focus on reduction of saturated fat intake. 50% of daily calories from carbohydrates; less than 10% from saturated fat and 20% from mono- and polyunsaturated fat, or up	**Primary outcome:**<br>Diabetes incidence<br><br>**Ancillary study of the Finnish Diabetes Prevention Study:**<br>CRP and IL-6

	conditions which could interfere with glucose metabolism.	Control: 86.0 (14.7)<br>**Mean BMI (SD) at baseline:**<br>Diet and exercise: 31.3 (4.6)<br>Control: 31.1 (4.6)<br>**IL-6 pg/ml baseline median (IR):**<br>Diet & exercise: 1.89 (1.15 – 2.85)<br>Control: 1.64 (1.09 – 2.51)<br>**TNF pg/ml baseline median (IQR):**<br>Diet & exercise: NA<br>Control: NA	to 25% if the surplus is from monounsaturated fat, 1.0 g protein per kg ideal body weight per. Increase in dietary fibre to 15 g per 1000 kcal or more.<br>***Calories:***<br>Personalised reduction in energy intake. If weight loss is not achieved during the first 6 to 12 months and the BMI is over 30 kg/m2, a very low calorie diet (VLCD) is considered for 6 to 12 weeks.<br>***Exercise:***<br>Individually guided to increase physical activity. Exercise programmes differ between study centres according to local situation and facilities. Supervised, progressive, individually tailored circuit-type resistance training sessions are organised, if possible, twice a week.<br><br>**Control:**<br>Verbal and written nutritionist information and advice to adjust total energy intake to reduce BMI below 25 kg/m2 and to keep to a diet with less than 30% of daily energy from fat. Advised to reduce alcohol intake and to stop smoking. Verbal general information about the health effects of recreational exercise is provided but no specific individual propositions and programmes are given. Routine advice at annual follow-up visits.	
**Imayama 2012, USA**[34]<br>An ancillary study of The Nutrition and Exercise for Women (NEW) study	**Inclusion criteria:**<br>Women, 50–75 years; BMI ≥ 25.0 kg/m2, <100 min/wk of moderate activity; postmenopausal; no hormone replacement therapy for the past 3 months; fasting glucose < 126 mg/dL; non-smoking; alcohol intake ≤2 drinks/d; able to attend facility; a normal exercise tolerance test.<br>**Exclusion criteria:**<br>History of breast cancer, heart disease, diabetes mellitus, or other serious medical conditions	**Participants (n)**<br>Diet: 118<br>Diet & exercise: 117<br>Exercise:117<br>Control: 87<br>**Sex (% female):**<br>All arms: % 100<br>**Mean age (SD) at baseline, years:**<br>Diet: 58.1 (6.0)<br>Diet & exercise: 58.0 (4.5)<br>Exercise: 58.1 (5.0)<br>Control arm: 57.4 (4.4)<br>**Mean weight (SD) at baseline, kg:**<br>Diet only: 84 (11.8)	**Arms:** Diet v's diet & exercise v's exercise v's control<br>**Duration:** 12 months<br>**Intervention:**<br>***Dietary advice and delivery:*** Participants had 2 to 4 Individual sessions with the dietitians, then met weekly in groups (5–10 women) until week 24, and afterward attended monthly group sessions in addition to e-mail or phone contacts.<br>***Recommended macronutrient content:***<br><30% fat<br>***Calories:***<br>Goal of caloric intake of 1,200 to 2,000 kcal/d based on weight, ≤30% calories from fat, aiming for 10% weight loss within the first 24 weeks, and maintenance thereafter<br>***Exercise:*** Diet & exercise and Exercise arms<br>Goal of 225 min/wk of moderate-to-vigorous intensity exercise. Attended 3 supervised sessions per week at the facility and 2 per week at home.	**Primary outcome:** serum Estrone<br><br>**Ancillary study of the NEW study:**<br>Inflammatory biomarkers

		Diet & exercise: 82.5 (10.8)<br>Exercise:83.7(12.3)<br>Control arm: 84.2 (12.5)<br>**Mean BMI (SD) at baseline:**<br>Diet only: 31.9 (3.9)<br>Diet & exercise: 31.0 (4.3)<br>Exercise:30.7 (3.7)<br>Control arm: 30.7 (3.9)<br>**IL-6 pg/ml baseline mean (SD):**<br>Diet only: 1.87 (1.77)<br>Diet & exercise: 1.53 (0.82)<br>Exercise: ?<br>Control arm: 1.69 (1.22)<br>**TNF pg/ml baseline:** NA	**Control:**<br>Asked not to change their diet or exercise habits.	
**Beavers 2013, USA**[29]	**Inclusion criteria:**<br>Age 60 to 79 years; BMI ≥ 28; evidence of a recent CVD incident or metabolic syndrome diagnosis, ≤ 60 minutes/week of moderate, structured PA<br><br>**Exclusion criteria:**<br>BMI ≥ 40 or higher; bipolar depression or schizophrenia; unstable angina, symptomatic congestive heart failure, or exercise-induced complex ventricular arrhythmias; blood pressure ≥ 160/100 mm Hg; diseases precluding from safely participating; fasting blood glucose level ≥140 mg/dL, type 1 diabetes mellitus, or T2DM with	**Participants (n)**<br>Diet & exercise: 98<br>Exercise: 97<br>**Sex (% female):**<br>Diet & exercise: 67 %<br>Exercise: 64 %<br>**Mean age (SD) at baseline, years:**<br>Diet & exercise: 66.8 (4.6)<br>Exercise: 67.2 (4.8)<br>**Mean weight (SD) at baseline, kg:**<br>Diet & exercise: 92.8 (16.1)<br>Exercise: 91.7 (13.1)<br>**Mean BMI (SD) at baseline:**<br>Diet & exercise: 33.1(4.1)<br>Exercise: 32.8 (3.9)<br>**IL-6 pg/ml baseline mean (SE):**<br>Diet & exercise: 2.9 (0.2)<br>Exercise: 2.6 (0.2)<br>**TNF pg/ml baseline: NA**	**Arms:** Diet & exercise vs exercise only v's successful aging health education<br>**Duration:**18 months<br>**Intervention:**<br>***Dietary advice and delivery:***<br>For the first 6 months, 3 group sessions (90 minutes) and one individual session (30 minutes) per month were conducted in a supervised setting. Months 7 to 18 formed a maintenance phase with 2 sessions per month. One contact was a group session and the second was a telephone contact that lasted approximately 10 to 20 minutes.<br>***Recommended macronutrient content:***<br>Recommendations for choices of foods were based on the US Department of Agriculture MyPyramid Food Guidance System focusing on low-fat and low-calorie foods.<br>***Calories:***<br>To reduce caloric intake to produce a WL of approximately 0.3 kg per week for the first 6 months for a total loss in mass of 7% to 10%. A 1200- to 1500-kcal goal was used for those weighing < 113.4 kg, and a 1500- to 1800-kcal goal for those weighing ≥ 113.4kg. During the weight maintenance phase, participants were encouraged to continue WL as long as their BMI was >20 kg/m2; however, the primary focus was weight maintenance.	**Primary outcome:** Time to complete a 400-m walk<br><br>**Ancillary study outcome:**<br>Biomarkers of inflammation

	insulin therapy; active treatment for cancer; clinically significant visual or hearing impairment; impaired cognitive function; ≥21 alcoholic drinks per week; inability to walk unassisted; inability to speak or read English.		***Exercise:***<br>For the first 6 months (intensive phase), 3 group sessions (90 minutes) and one individual session (30 minutes) per month in a supervised setting. Months 7 to 18 (maintenance phase), two session per month. One contact was a group session and the second was a telephone contact that lasted approximately 10 to 20 minutes. Participants were asked to walk for 30 minutes on most days of the week.<br><br>**Control/Successful aging health education**<br>18 sessions included health topics relevant to older adults such as how the body changes with aging, prevention or delaying disease, eating for good health, positive attitudes toward aging, family relationships and care giving, and talking to health care providers	
**Messier 2013, USA**[32]	**Inclusion criteria:**<br>Age ≥ 55 years with mild to moderate radiographic tibiofemoral OA or tibiofemoral plus patellofemoral OA of one or both knees, pain on most days due to knee OA, BMI ≥ 27.0 and ≤ 41 kg/m2; not more than 30 minutes per week of formal exercise within the past 6 month<br>**Exclusion criteria:**<br>Significant co-morbid disease impairing ability to participate, previous acute knee injury, patellofemoral OA in the absence of tibiofemoral OA, unwillingness or inability to change eating and physical activity habits due to environment; unable to read	**Participants (n)**<br>Diet & exercise: 152<br>Exercise: 150<br>**Sex (% female):**<br>Diet & exercise: 72 %<br>Exercise: 72 %<br>**Mean age (SD) at baseline, years:**<br>Diet & exercise: 65 (6)<br>Exercise: 66 (6)<br>**Mean weight (SD) at baseline, kg:**<br>Diet & exercise: 93 (14.4)<br>Exercise: 92 (14.5)<br>**Mean BMI (SD) at baseline:**<br>Diet & exercise: 33.6 (3.7)<br>Exercise: 33.5 (3.7)<br>**IL-6 pg/ml baseline mean (SD):**<br>Diet & exercise: 3.2 (2.22)<br>Exercise: 3 (2.1)<br>**TNF pg/ml baseline: NA**	**Arms:** Diet v's diet & exercise v's exercise<br>**Duration:** 18 months<br>***Dietary advice and delivery:***<br>Aim for 10% body weight loss. 1 individual session and 3 group sessions per month for 6 months, then biweekly group sessions and an individual session every 2 months until 18 months.<br>***Recommended macronutrient content:***<br>15% to 20% from protein, less than 30% from fat, and 45% to 60% from carbohydrates.<br>***Calories:***<br>Initially two meal-replacement shakes per day and a meal low in fat, and high in vegetables, reducing shakes and increasing meals over time. Daily caloric intake was adjusted according to the rate of weight change between intervention visits. Energy-intake deficit of 800 to 1000 kcal/day as predicted by energy expenditure with at least 1100 kcal for women and 1200 kcal for men.<br>***Exercise: both groups***<br>1 hour on 3 days/ week for 18 months consisting of aerobic and strength training. During the first 6 months, participation was centre-based. After 6-month follow-up testing and a 2-week transition phase, participants could remain in the facility program, opt for a home-based program, or combine the two. The program	**Primary outcomes-** Knee joint compressive force and plasma IL-6 levels.

	English, excess alcohol use, conditions that prohibit knee MRI, significant cognitive impairment or depression		consisted of aerobic walking (15 minutes), strength training(20minutes), a second aerobic phase (15 min- utes), and cool-down (10 minutes).	
**Miller 2014, USA**[31]	**Inclusion criteria:**<br>≥ 21 years of age, BMI ≥ 25 to ≤ 40 kg/m^2); blood glucose of 95 mg/dl to 125 mg/dl following at least an 8-hour fast; no medical contraindications to participate in a lifestyle intervention<br>**Exclusion criteria:**<br>Diabetes, recent history of cardiovascular disease, uncontrolled HTN behavioural or psychiatric conditions.	**Participants (n)**<br>Diet and exercise: 151<br>Control: 150<br>**Sex (% female):**<br>Diet & exercise: 70%<br>Control: 70%<br>**Mean age (SD) at baseline, years:**<br>Diet & exercise: 60.3 (9.8)<br>Control: 54.9 (7.2)<br>**Mean weight (SD) at baseline, kg:**<br>Diet & exercise: 94.4 (14.7)<br>Control: 93 (16.2)<br>**Mean BMI (SD) at baseline:**<br>Diet & exercise: 33.0 (3.2)<br>Control: 34.2 (4.1)<br>**IL-6 pg/ml baseline mean (95% CI):**<br>Diet & exercise, n=15: 0.3 (0.2 to 0.5)<br>Control, n=15: 0.3 (0.2 to 0.6)<br>**TNF pg/ml baseline mean (95% CI):**<br>Diet & exercise, n=15: 14.3 (11.4 to 17.8)<br>Control, n=15: 12.8 (11 to 14.9)	**Arms:** Diet and exercise v's Control<br>**Duration:**12 months<br>**Intervention:**<br>***Dietary advice and delivery:***<br>Months 1 to 6: Weekly group sessions with Community Health Workers (CHW) and three individual sessions with a dietician.<br>Months 7 to 12: 1 group session and one phone contact with a CHW per month.<br>***Recommended macronutrient content:***<br>Non-specific 'Healthy eating'<br>***Calories:***<br>Targeted decrease in calorie intake, goal of 1200–1800 kcal/day to produce a weight loss of approximately 0.3 kg per week for the first 6-months, followed by either maintenance or continued weight loss as long as BMI does not fall below 20 kg/m2.<br>***Exercise:***<br>A goal of > 180 min/wk of moderate physical activity (not clear if supervised)<br>**Control:**<br>Enhanced prediabetes usual care—2 individual sessions with a nutritionist in months 0–3 covering basic healthy eating and activity for weight loss, followed by a quarterly newsletter with topics related to a healthy lifestyle.	**Primary study outcome:** Change in Fasting blood glucose and insulin resistance<br><br>**Ancillary study:** Adipose Tissue Generated Mediators of Cardiovascular Risk

**Thompson 2014, UK**[35]	**Inclusion criteria:** Age > 30 years at diagnosis of T2D within the previous 5 to 8 months<br><br>**Exclusion criteria:**<br>Age > 80 years, HbA1c > 10%, BP > 180/100 mm Hg, LDL > 4 mmol/L, BMI < 25 kg/m2, Weight > 180 kg, use of weight-loss drugs, maximum dose sulphonylurea, unstable angina, MI within the previous 3 months, inability to increase physical activity, pregnancy or planned pregnancy.	**Participants (n)**<br>Diet only: 248<br>Control arm: 99<br>**Sex (% female):**<br>Diet only: 36%<br>Control: 37%<br>**Mean age (SD) at baseline, years:**<br>Diet only: 60 (10)<br>Control: 60 (11)<br>**Mean weight (SD) at baseline, kg:**<br>Diet only: 90.2 (16.7)<br>Control: 93.9 (19.0)<br>**Mean BMI (SD) at baseline:**<br>Diet only: 31.5 (5.7)<br>Control: 32.3 (5.9)<br>**IL-6 pg/ml baseline geometric mean (SD)**<br>Diet only: 2 (1.9)<br>Control: 2.4 (1.9)<br>**TNF pg/ml baseline:** NA	**Arms:** Diet v's Diet & exercise v's Control<br>**Duration:** 12 months<br>**Intervention:**<br>***Dietary advice and delivery:***<br>Intensive diet intervention to enable weight loss of 5% to 10% of initial body weight. Participants saw a dietitian at 3, 6, 9, and 12 months, supplemented by dietary advice and goal setting with nurses by nine 30-minute appointments approximately every 6 weeks.<br>***Recommended macronutrient content:***<br>The diet was not prescriptive;<br>***Calories:***<br>Goals were negotiated individually with each participant.<br>***Exercise: Diet and exercise arm only***<br>To undertake at least 30 minutes of brisk walking on at least 5 days per week over and above their existing physical activity.<br><br>**Control:**<br>Usual care standard dietary and exercise advice by a study doctor and nurse at baseline, 6 and 12 months	**Primary outcome:**<br>HbA1c and BP<br><br>**Ancillary study outcome:**<br>Systemic markers of inflammation
**Welsh 2016, Scotland**[36]	**Inclusion criteria:**<br>Pakistani or Indian ethnic origin, aged ≥ 35 years, impaired glucose tolerance or impaired fasting glucose according to WHO criteria.<br><br>**Exclusion criteria:**<br>Diabetes, long-term oral corticosteroids, weight loss medication, health disorders making adherence contraindicated or improbable, pregnant, or who	**Participants (n)**<br>Diet & exercise: 58<br>Control: 76<br>**Sex (% female):**<br>Diet & exercise: 66<br>Control: 55<br>**Mean age (SD) at baseline, years:**<br>Diet & exercise: 52.6 (10.3)<br>Control: 52.4 (9.8)<br>**Mean weight (SD) at baseline, kg:**<br>Diet & exercise: 79.2 (16.7)<br>Control: 80.5 (15.5)<br>**Mean BMI (SD) at baseline:**	**Arms:** Diet & exercise v's Control<br>**Duration:** 3 years<br>**Intervention:**<br>***Dietary advice and delivery:***<br>15 visits from a dietitian over 3 years, (baseline, monthly for the first 3 months, then every 3 months) who advised on achieving weight loss through calorie deficit using culturally sensitive techniques.<br>***Recommended macronutrient content:***<br>None specific<br>***Calories:***<br>None specific calorie deficit<br>***Exercise:***<br>Physical activity of at least 30 min daily brisk walking.	**Primary outcome**-Weight change<br><br>**Planned analysis of:**<br>Cardiometabolic risk profile: lipids, liver function and inflammatory and metabolic markers

	were unlikely to remain in the UK for 3 years.	Diet & exercise: 30.5 (5.2)<br>Control: 30.5 (4.8)<br>**IL-6 pg/ml baseline median (IR):**<br>Diet & exercise: 1.15 (0.76 – 2.09)<br>Control: 1.28 (0.83 – 2.14)<br>**TNF pg/ml baseline: NA**	**Control:**<br>Standardized written and verbal advice on healthy eating, diabetes prevention, promotion of physical activity and on accessing other weight control and physical activity services over four visits (baseline, then annually) with a dietitian.	
**Ard 2017, USA** [33]	**Inclusion criteria:**<br>Aged ≥ 65 years, weight stable, BMI ≥ 30 to ≤ 40 kg/m^2; prescribed at least one oral medication for control of lipids, blood pressure, and/or blood glucose; capacity to be actively involved in treatment.<br><br>**Exclusion criteria:**<br>BMI ≥ 40 kg/m^2, significant medical, psychiatric, or physical limitations that would prevent adoption of the lifestyle recommendations or ongoing treatments that would independently affect body weight and composition.	**Participants (n)**<br>Diet and exercise: 55<br>Exercise only: 54<br>**Sex (% female):**<br>Diet & exercise: 48.2<br>Exercise only 68.5<br>**Mean age (SD) at baseline, years:**<br>Diet & exercise: 70.3 (4.8)<br>Exercise only: 69.9 (4.5)<br>**Mean weight (SE) at baseline, kg:**<br>Diet & exercise: 94.1 (2.1)<br>Exercise only: 95.2 (1.7)<br>**Mean BMI (SE) at baseline:**<br>Diet & exercise: 33.3 (0.4)<br>Exercise only: 33.9 (0.4)<br>**IL-6 pg/ml baseline mean (SE):**<br>Diet & exercise: 2.4 (0.2)<br>Exercise only: 3 (0.4)<br>**TNF pg/ml baseline mean (SE):**<br>Diet & exercise: 4.7 (0.2)<br>Exercise only: 5.4 (0.2)	**Arms:** Dietary weight loss and exercise v's exercise only v's weight maintenance and exercise<br>**Duration:** 12 months<br>**Intervention:**<br>***Dietary advice and delivery:***<br>All groups received dietician led group behavioural counselling weekly for 24 weeks, then every 2 weeks. Recommendations to increase low-energy dense fruits, vegetables, lean protein, and whole grains.<br>***Recommended macronutrient content:***<br>25% protein, 47% carbohydrates, 28% f fat.<br>***Calories:***<br>Reduced caloric intake by 500 kcal/d below estimated total energy needs based on measured resting energy expenditure, with a minimum intake of 1,000 kcal/d<br>***Exercise (all groups):***<br>Recommendations for 90–150 min/ wk of moderate to vigorous cardio-aerobic exercise. All group sessions were followed by 30 minutes of supervised exercise using resistance band exercises. Participants also received a written program to guide participation in two sessions/ week of resistance training.	**Primary outcome:** change in visceral adipose tissue on MRI.<br><br>**Secondary outcomes:** Cardiometabolic risk biomarkers, functional status, QOL
**Serra-Prat 2022, Spain** [28]	**Inclusion criteria:**<br>Aged 65-75 years, with a BMI of 30-39 kg/m^2	**Participants (n)**<br>Diet & exercise: 150<br>Control: 155<br>**Sex (% female):**	**Arms:** Diet & exercise v's Control<br>**Duration:** 6 month intervention, outcomes at 6, 12 and 24 months<br>**Intervention:**<br>***Dietary advice and delivery:***	**Primary outcomes:** Prevalence of frailty.

	(inclusive), with least one of the following: dyslipidaemia, HTN, diabetes or insulin resistance, obesity-related physical limitations, or sleep apnoea/hypopnoea<br><br>**Exclusion criteria:**<br>Dementia, neurodegenerative diseases, severe psychiatric disorders, cancer, lower limb amputation, institutionalization, and life expectancy <6 months.	Diet & exercise: 69.3<br>Control: 62.6<br>**Mean age (SD) at baseline, years:**<br>Diet & exercise: 69.6 (2.7)<br>Control: 69.9 (2.7)<br>**Mean weight (SD) at baseline, kg:**<br>Diet & exercise: not available<br>Control: not available<br>**Mean BMI (SD) at baseline:**<br>Diet & exercise: 34.2 (3.3)<br>Control: 34.0 (3.2)<br>**IL-6 pg/ml baseline mean (SD)**<br>Diet & exercise: 55 (17)<br>Control: 4.5 (11.2)<br>**TNF pg/ml baseline:** NA	A dietician personalized eating plan aimed at achieving BMI<30 or weight loss>10% within 6 months. Monthly individualized sessions with a dietician.<br>***Recommended macronutrient content:***<br>20% protein (1.2 g/kg/day), 50% carbohydrates, 27% fat<br>***Calories:***<br>Caloric deficit of 300-400 kcal/day with respect to the daily energy expenditure DEE<br>***Exercise:***<br>Home based unsupervised strength, balance, and flexibility exercises for 15-20 minutes/day on 3 days a week, and health education by a physiotherapist, consisting of 20 theoretical- practical group sessions of 1 hour/week in the primary care centre, aimed at improving adherence and emphasizing the importance of physical exercise and also including Nordic walking in groups twice a month.<br><br>**Control:**<br>Usual care including standard primary care dietary and hygienic recommendations.	**Intermediate outcomes** .<br>Weight loss, BMI, body fat distribution, metabolic and inflammatory biomarker changes.

Figure S3: Quality assessment of all RCT's

Risk of Bias Assessment

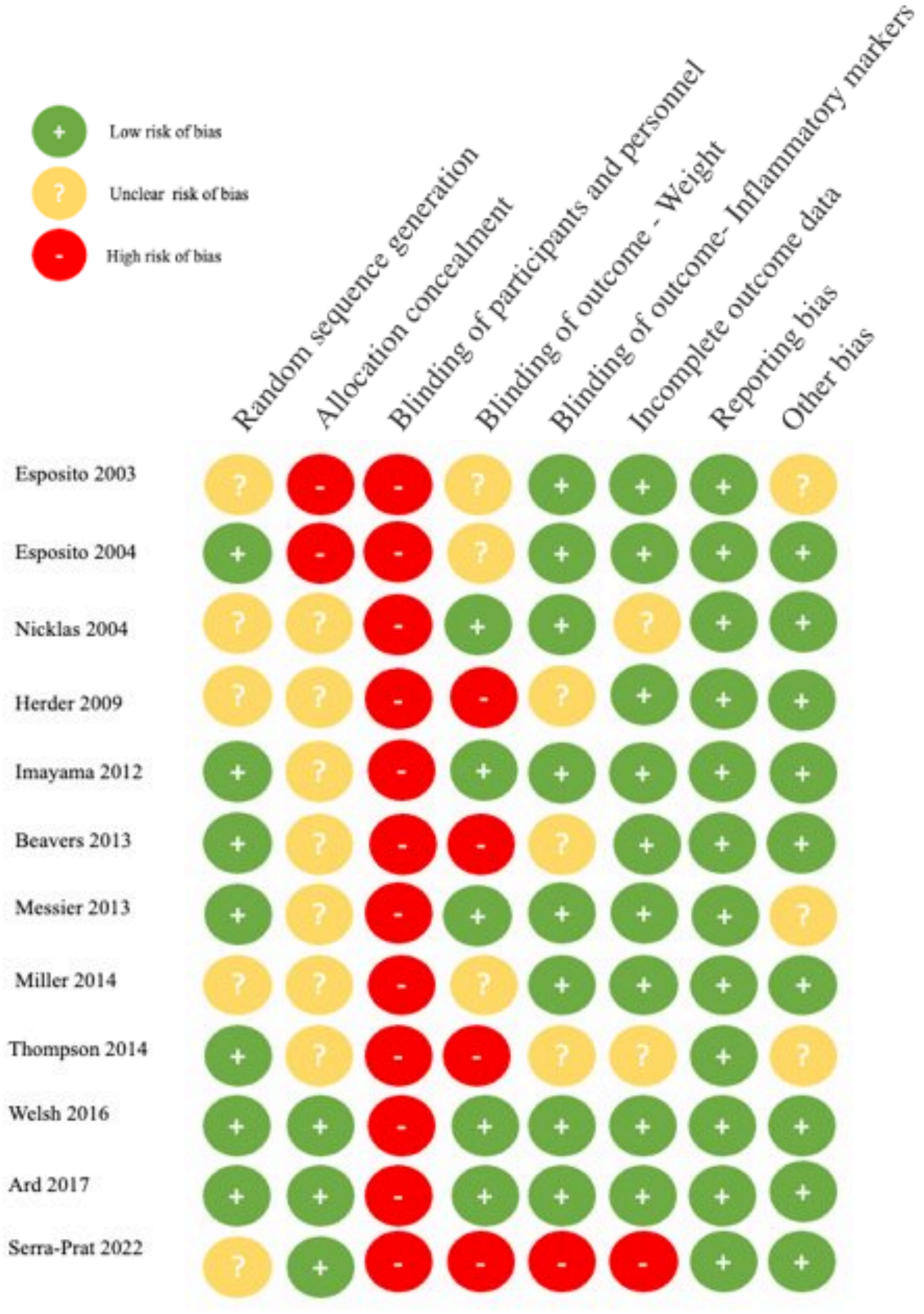

Figure S4: **Funnel plot: Change in weight subgroups**

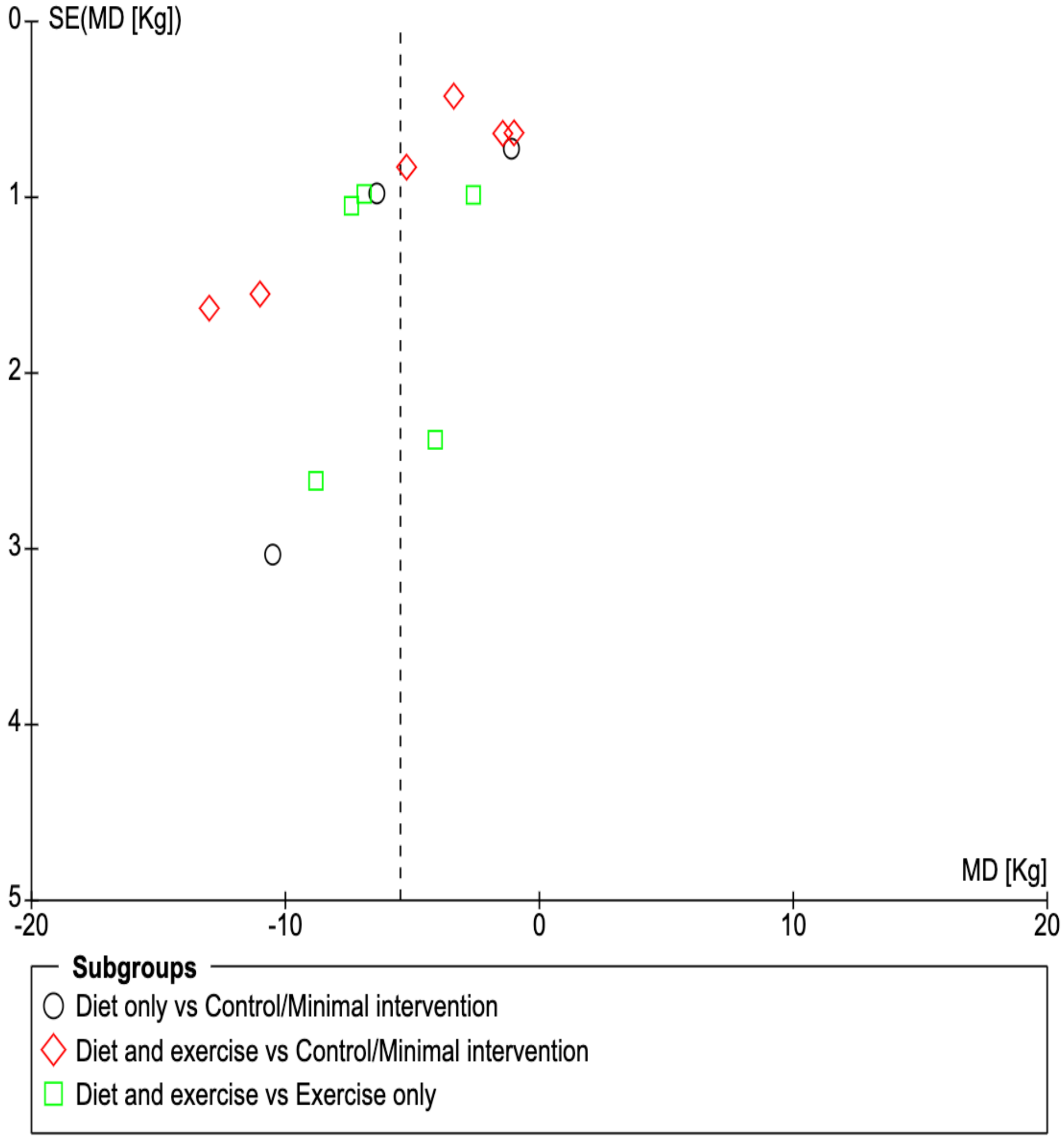

Figure S5: Forest plot.

Change in weight: Effect of intervention versus control on weight change in kg

Study or Subgroup	Intervention Mean [Kg]	Intervention SD [Kg]	Intervention Total	Control Mean [Kg]	Control SD [Kg]	Control Total	Weight	Mean difference IV, Random, 95% CI [Kg]
**1.1.1 Diet only v Control/Minimal intervention**								
Nicklas 2004. a:Diet v's Control	-12.8	19.2	53	-2.3	11.6	60	4.5%	-10.50 [-16.44 , -4.56]
Imayama 2012. a:Diet v's Control	-7.1	7.9	118	-0.7	6.1	87	8.5%	-6.40 [-8.32 , -4.48]
Thompson 2014	-1.5	6.3	248	-0.4	6	99	8.9%	-1.10 [-2.52 , 0.32]
**Subtotal (95% CI)**			**419**			**246**	**21.9%**	**-5.38 [-10.18 , -0.59]**
Heterogeneity: Tau² = 15.15; Chi² = 24.91, df = 2 (P < 0.00001); I² = 92%								
Test for overall effect: Z = 2.20 (P = 0.03)								
**1.1.2 Diet and Exercise v Exercise only**								
Nickals 2004. b: Diet and exercise v's Exercise	-8.2	13.3	53	-4.1	11.1	53	5.6%	-4.10 [-8.76 , 0.56]
Imayama 2012. b: Diet and exercise v's Exercise	-8.9	8.4	117	-2	6.48	117	8.5%	-6.90 [-8.82 , -4.98]
Beavers 2013	-7.1	7.92	98	-0.8	6.14	97	8.4%	-6.30 [-8.29 , -4.31]
Messier 2013	-10.6	19.6	121	-1.8	20.5	115	5.2%	-8.80 [-13.92 , -3.68]
Ard 2017	-3.9	5.2	55	-1.3	5.1	54	8.5%	-2.60 [-4.53 , -0.67]
**Subtotal (95% CI)**			**444**			**436**	**36.2%**	**-5.52 [-7.67 , -3.37]**
Heterogeneity: Tau² = 3.77; Chi² = 13.16, df = 4 (P = 0.01); I² = 70%								
Test for overall effect: Z = 5.03 (P < 0.00001)								
**1.1.3 Diet and Exercise v Control/Minimal intervention**								
Esposito 2003	-14	9.9	60	-3	6.8	60	7.3%	-11.00 [-14.04 , -7.96]
Esposito 2004	-15	10.2	55	-2	6.5	55	7.2%	-13.00 [-16.20 , -9.80]
Herder 2009	-4.63	4.88	207	-1.26	3.6	199	9.3%	-3.37 [-4.20 , -2.54]
Welsh 2016	-1.14	4.2	75	0.3	3.6	76	9.0%	-1.44 [-2.69 , -0.19]
Serra-Prat 2022	-2.7	3.5	77	-1.7	4.8	95	9.1%	-1.00 [-2.24 , 0.24]
**Subtotal (95% CI)**			**474**			**485**	**41.9%**	**-5.52 [-8.48 , -2.56]**
Heterogeneity: Tau² = 10.32; Chi² = 80.93, df = 4 (P < 0.00001); I² = 95%								
Test for overall effect: Z = 3.65 (P = 0.0003)								
**Total (95% CI)**			**1337**			**1167**	**100.0%**	**-5.41 [-7.14 , -3.68]**
Heterogeneity: Tau² = 8.17; Chi² = 133.21, df = 12 (P < 0.00001); I² = 91%								
Test for overall effect: Z = 6.14 (P < 0.00001)								
Test for subgroup differences: Chi² = 0.00, df = 2 (P = 1.00), I² = 0%								

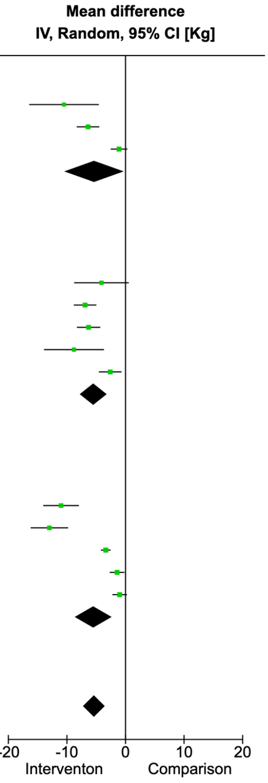

Figure S6: Forest plot sensitivity analysis- Blinding of weight loss outcome

Study or Subgroup	Intervention Mean [Kg]	Intervention SD [Kg]	Intervention Total	Control Mean [Kg]	Control SD [Kg]	Control Total	Weight	Mean difference IV, Random, 95% CI [Kg]	A	B	C	D	E	F	G
**1.1.1 Diet only v Control/Minimal intervention**															
✔ Nicklas 2004. a:Diet v's Control	-12.8	19.2	53	-2.3	11.6	60	8.2%	-10.50 [-16.44 , -4.56]	?	?	−	+	?	+	+
✔ Imayama 2012. a:Diet v's Control	-7.1	7.9	118	-0.7	6.1	87	12.5%	-6.40 [-8.32 , -4.48]	+	?	−	+	+	+	+
✖ Thompson 2014	-1.5	6.3	248	-0.4	6	99	0.0%	-1.10 [-2.52 , 0.32]	+	?	−	−	?	+	?
**Subtotal (95% CI)**			**171**			**147**	**20.7%**	**-7.44 [-10.95 , -3.94]**							
Heterogeneity: Tau² = 3.33; Chi² = 1.66, df = 1 (P = 0.20); I² = 40%															
Test for overall effect: Z = 4.17 (P < 0.0001)															
**1.1.2 Diet and Exercise v Exercise only**															
✔ Nickals 2004. b: Diet and exercise v's Exercise	-8.2	13.3	53	-4.1	11.1	53	9.6%	-4.10 [-8.76 , 0.56]							
✔ Imayama 2012. b: Diet and exercise v's Exercise	-8.9	8.4	117	-2	6.48	117	12.5%	-6.90 [-8.82 , -4.98]							
✖ Beavers 2013	-7.1	7.92	98	-0.8	6.14	97	0.0%	-6.30 [-8.29 , -4.31]	+	?	−	−	+	+	+
✔ Messier 2013	-10.6	19.6	121	-1.8	20.5	115	9.1%	-8.80 [-13.92 , -3.68]	+	?	−	+	+	+	?
✔ Ard 2017	-3.9	5.2	55	-1.3	5.1	54	12.5%	-2.60 [-4.53 , -0.67]	+	+	−	+	+	+	+
**Subtotal (95% CI)**			**346**			**339**	**43.7%**	**-5.33 [-8.26 , -2.40]**							
Heterogeneity: Tau² = 6.05; Chi² = 11.92, df = 3 (P = 0.008); I² = 75%															
Test for overall effect: Z = 3.57 (P = 0.0004)															
**1.1.3 Diet and Exercise v Control/Minimal intervention**															
✔ Esposito 2003	-14	9.9	60	-3	6.8	60	11.4%	-11.00 [-14.04 , -7.96]	?	−	−	?	+	+	?
✔ Esposito 2004	-15	10.2	55	-2	6.5	55	11.3%	-13.00 [-16.20 , -9.80]	+	−	−	?	+	+	+
✖ Herder 2009	-4.63	4.88	207	-1.26	3.6	199	0.0%	-3.37 [-4.20 , -2.54]	?	?	−	−	+	+	+
✔ Welsh 2016	-1.14	4.2	75	0.3	3.6	76	12.9%	-1.44 [-2.69 , -0.19]	+	+	−	+	+	+	+
✖ Serra-Prat 2022	-2.7	3.5	77	-1.7	4.8	95	0.0%	-1.00 [-2.24 , 0.24]	?	+	−	−	−	+	+
**Subtotal (95% CI)**			**190**			**191**	**35.7%**	**-8.38 [-16.54 , -0.22]**							
Heterogeneity: Tau² = 50.19; Chi² = 66.99, df = 2 (P < 0.00001); I² = 97%															
Test for overall effect: Z = 2.01 (P = 0.04)															
**Total (95% CI)**			**707**			**677**	**100.0%**	**-6.95 [-9.69 , -4.20]**							
Heterogeneity: Tau² = 14.75; Chi² = 87.29, df = 8 (P < 0.00001); I² = 91%															
Test for overall effect: Z = 4.96 (P < 0.00001)															
Test for subgroup differences: Chi² = 1.08, df = 2 (P = 0.58), I² = 0%															

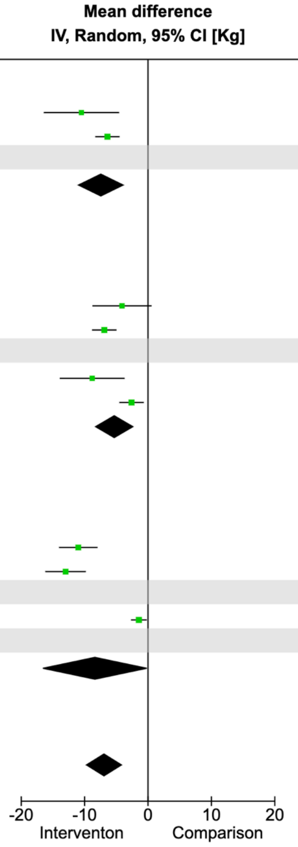

Risk of bias legend
(A) Random sequence generation (selection bias)
(B) Allocation concealment (selection bias)
(C) Blinding of participants and personnel (performance bias)
(D) Blinding of outcome assessment (detection bias)
(E) Incomplete outcome data (attrition bias)
(F) Selective reporting (reporting bias)
(G) Other bias

Table S2:

Effect of dietary weight loss intervention studies on weight loss (kg) and serum inflammatory markers (%)

	Comparison arms, n=	Mean age (SD), % female	Participant characteristic	Baseline weight kg (SD)	Intervention	Within group weight loss (kg) (between group p value)	Within group % change in IL-6 (between group p value)	Within group % change TNF-α(between group p value)
Diet v's Standard care/minimal intervention								
**Nicklas[30] 2004: a**	*Diet n=53*<br>*Control n=60*	68 (5), 74<br>69 (6), 66	OA	95.6 (152)<br>95.7 (18.8)	CR diet<br>18 months	-12.8<br>-2.3<br>P<0.0001	-15<br>+6<br>P=0.009	+26<br>-21<br>P=0.67
**Imayama[34] 2012: a**	*Diet n=118*<br>*Control n=87*	58.1 (6.0), 100<br>57.4 (4.4), 100	Postmenopausal	84 (11.8)<br>84.2 (12.5)	CR diet<br>12 months	-7.1<br>-0.7<br>P<0.0001	-14<br>+12<br>P=0.001	NA
**Thompson[35] 2014**	*Diet n=248*<br>*Control n=99*	60 (10), 36<br>60 (11), 37	Newly diagnosed T2DM	90.2 (16.7)<br>93.9 (19.0)	CR diet<br>12 months	-1.5<br>-0.4<br>P<0.0001	-8<br>-11<br>NA	NA
Diet and exercise v's Standard care/minimal intervention								
**Esposito[38] 2003**	*Diet & exercise n=60*<br>*Control n=60*	34.2, 100<br>35, 100	Pre-menopausal	95 (9.4)<br>94 (9.2)	CR Mediterranean diet<br>24 months	-14<br>-3<br>P<0.0001	-32<br>-7<br>P=0.009	NA
**Esposito[37] 2004**	*Diet & exercise n=55*<br>*Control n=55*	43.5 (4.8), 0<br>43 (5.1), 0	Erectile dysfunction	103 (9.4)<br>101 (9.7)	CR Mediterranean diet<br>24 months	-15<br>-2<br>P=0.007	-31<br>+2<br>P=0.03	NA
**Herder[39] 2009**	*Diet & exercise n=207*<br>*Control n=199*	55.7 (7.1), 64<br>55.1 (6.9), 66	IGT	86.9 (14.2)<br>86.0 (14.7)	CR diet<br>12 months	-4.6<br>-1.3<br>P<0.001	-17<br>+0.6<br>P=0.033	NA
**Miller[31] 2014**	*Diet & exercise n=151*<br>*Control n=150*	60.3 (9.8), 70<br>54.9 (7.2), 70	IGT	94.4 (14.7)<br>93 (16.2)	CR diet<br>12 months	-7<br>-2<br>P<0.001	-33<br>+33<br>P=0.001	-6<br>-17<br>P=0.083
**Welsh[36] 2016**	*Diet & exercise n=75*<br>*Control n=76*	52.6 (10.3), 66<br>52.4 (9.8), 55	Pakistani or Indian ethnic origin, IGT	79.2 (16.7)<br>80.5 (15.5)	CR diet<br>36 months	-1.1<br>+0.3<br>P=0.026	-21<br>-19<br>P=0.899	NA

**Serra-Pratt**[28] **2022**	*Diet & exercise n=77*<br>*Control n=95*	69.6 (2.7), 69<br>69.9 (2.7), 63	Features of metabolic syndrome and/or obesity-related hypoventilation syndromes	Not stated	CR diet<br>24 months	-2.7<br>-1.7<br>P>0.05	-27<br>+24<br>P=0.221	NA
Diet and exercise v's exercise only								
**Nicklas**[30] **2004: b**	*Diet & exercise n=53*<br>*Exercise n=53*	68 (7), 74<br>69 (6), 74	OA	91.8 (17.4)<br>92.4 (14.6)	CR diet<br>18 months	-8.2<br>-4.1<br>NA	-7<br>+0.5<br>NA	-21<br>+8<br>NA
**Imayama**[34] **2012: b**	*Diet & exercise n=117*<br>*Exercise n=117*	58.0 (4.5), 100<br>58.1 (5.0), 100	Postmenopausal	82.5 (10.8)<br>83.7(12.3)	CR diet<br>12 months	-8.9<br>-2<br>P<0.0001	-16<br>+7<br>P<0.001	NA
**Beavers**[29] **2013**	*Diet & exercise n=98*<br>*Exercise n=97*	66.8 (4.6), 67<br>67.2 (4.8), 64	recent CVD incident or metabolic syndrome diagnosis	92.8 (16.1)<br>91.7 (13.1)	CR diet<br>18 months	-8.3<br>-0.9<br>P<0.001	-14<br>+4<br>P<0.05	NA
**Messier**[32] **2013**	*Diet & exercise n=152*<br>*Exercise n=150*	65 (6), 72<br>66 (6), 72	OA	93 (14.4)<br>92 (14.5)	CR diet<br>18 months	-10.6<br>-1.8<br>P<0.001	-15<br>0<br>P=0.007	NA
**Ard**[33] **2017**	*Diet & exercise n=55*<br>*Exercise n=54*	70.3 (4.8), 48<br>69.9 (4.5), 69	≥ 1 medication for hyperlipidaemia, HTN or diabetes	94.1 (2.1)*<br>95.2 (1.7)*	CR diet<br>12 months	-3.9<br>-1.3<br>P<0.01	-13<br>-3<br>P=0.111	-2<br>+9<br>P=0.048

Reached significance	Reached significance
Did not reach significance	Did not reach significance

Abbreviations: CR: Calorie restriction, **OA**: Osteoarthritis, **T2DM**: Type II Diabetes mellitus, **IGT**: Impaired glucose tolerance, **CVD**: cardiovascular, **HTN**: Hypertension, **IL-6**: Interleukin 6, **TNF-α**: Tumour necrosis factor, **NA**: Not available

* **SE**

Table S3. Effect of dietary weight loss intervention on percentage change weight and serum inflammatory markers over time

			Within group Weight % and IL-6 % change from baseline at:				
			**6 months**	**12 months**	**18 months**	**24 months**	
**Welsh [36] 2016**	***Diet & exercise n=75***	Weight %					-1.4
		IL-6 %					*-21*
	Control n=76	Weight %					+0.4
		IL-6 %					*-19*
**Esposito [38] 2003**	***Diet & exercise n=60***	Weight %				-15	
		IL-6 %				*-33*	
	Control n=60	Weight %				-3	
		IL-6 %				*-7*	
**Esposito [37] 2004**	***Diet & exercise n=55***	Weight %				-15	
		IL-6 %				*-31*	
	Control n=55	Weight %				-2	
		IL-6 %				*+2*	
**Herder [39] 2009**	***Diet & exercise n=207***	Weight %		-5.3			
		IL-6 %		*-17*			
	Control n=199	Weight %		-1			
		IL-6 %		*+0.6*			
**Miller[31] 2014**	***Diet & exercise n=151***	Weight %		-7.8			
		IL-6 %		*-33*			
	Control n=150	Weight %		-1.7			
		IL-6 %		*+33*			
**Messier[32] 2013**	***Diet & exercise n=152***	Weight %	-9			-11.4	
		IL-6 %	-13			-15	
	Exercise n=150	Weight %	+0.1			-2	
		IL-6 %	-3			*0*	
**Ard [33] 2017**	*Diet & exercise n=55*	Weight %	-4	-4			
		IL-6 %	-8	-13			

	Exercise n=54	Weight %	- 1.3	-1.4			
		IL-6 %	-3	+3			
**Thompson[35] 2014**	***Diet n=248***	Weight %	-1.8	-1.6			
		IL-6 %	-3	-8			
	Control n=99	Weight %	+0.3	-0.4			
		IL-6 %	-10	-11			
**Imayama[34] 2012: a**	***Diet n=118***	Weight %		-8.5			
		IL-6 %		-14			
	Control n=87	Weight %		-0.8			
		IL-6 %		+12			
**Imayama[34] 2012: b**	*Diet & exercise n=117*	Weight %		-10.8			
		IL-6 %		-16			
	Exercise n=117	Weight %		-2.4			
		IL-6 %		+7			
**Serra-Prat[28] 2022**	*Diet & exercise n=77*	Weight*	-4.3 (4.1)	-3.3 (4.2)			-2.7(3.5)
		IL-6 %	NA	-25			-27
	Control n=95	Weight*	-1.1(3.3)	-0.5(4.1)			-1.7(4.8)
		IL-6 %	NA	+26			+24
**Beavers[29] 2013**	*Diet & exercise n=98*	Weight %	-9		-7.7		
		IL-6 %	-3		-14		
	Exercise n=97	Weight %	-0.5		-1		
		IL-6 %	+11		+4		
**Nicklas[30] 2004: a**	***Diet n=53***	Weight %	-11			-13	
		IL-6 %	-10			-15	
	Control n=60	Weight %	-0.1			-2.4	
		IL-6 %	+4			+6	
**Nicklas[30] 2004: b**	*Diet & exercise n=53*	Weight %	-11			-9	
		IL-6 %	-7			-7	
	Exercise n=53	Weight %	-3			-4.4	
		IL-6 %	+3			+0.5	

*Mean Kg (SD) (percentage change not available)

Intervention phase	Maintenance phase
